# Pupil- and gaze dynamics track emotion content in naturalistic speech

**DOI:** 10.1038/s41598-026-57256-0

**Published:** 2026-06-18

**Authors:** Sümeyye Şen-Alpay, Christian Keitel, Rosanne H. Timmerman, Anne Keitel

**Affiliations:** 1 Psychology, University of Dundee, Dundee, UK; 2 CNNP Lab, School of Computing, Newcastle University, Newcastle upon Tyne, UK NE45TG

**Keywords:** Speech perception, Active sensing, Emotion, Naturalistic speech, Pupillometry, Eye movements, Neuroscience, Psychology, Psychology

## Abstract

Cortical tracking of speech features is a well-established marker of continuous speech processing, but far less is known about listeners’ ocular responses to speech rhythm. Ocular responses are central to active sensing models in the auditory domain, where motor recruitment guides temporal speech prediction and attention allocation, potentially shaped by non-rhythmic cues, such as emotions. Here, we ask whether listeners’ pupil response and eye movements track the acoustic speech signals and to what extent this tracking is modulated by emotion-related top-down factors, including subjective emotion ratings, mood, and trait empathy. In a validation study (*N* = 100), participants passively listened to two TED talks and intermittently rated speech segments on valence (negative–positive) and arousal (low–high). This suggested substantial variability in valence and arousal across speech segments in both talks. In the second study (*N* = 41), participants completed the same task while pupillometry and electrooculography (EOG) were recorded. Mutual information was used to quantify speech tracking in pupil dilation, along with horizontal and vertical eye movements. All ocular signals significantly tracked speech at low frequencies. High-arousal speech was associated with stronger pupil tracking but weaker vertical and horizontal EOG tracking. Negative speech valence was linked to stronger tracking in pupil and vertical eye-movement signals. Interactions between the speech-emotion dimensions, as well as their interactions with listeners’ mood, further shaped these effects, giving rise to distinct patterns across ocular measures. Taken together, our findings provide evidence that ocular activity dynamically aligns to the temporal structure of natural speech and that this tracking is sensitive to both stimulus-driven and listener-dependent emotional factors.

**Supplementary Information:**

The online version contains supplementary material available at https://doi.org/10.1038/s41598-026-57256-0.

## Introduction

Speech is a complex, dynamic acoustic signal that conveys both linguistic and paralinguistic information across time and frequency. Its real-time perception depends on the brain’s ability to parse and interpret these spectro-temporal patterns. Over the past decade, this challenge has driven auditory cognitive neuroscience, with oscillation-based models highlighting speech tracking as a potential mechanism in naturalistic contexts^1–3^. In these models, the quasi-rhythmic temporal structure of speech is highlighted as a core feature in the neural processing of speech. This quasi-rhythmicity in speech is typically quantified by the speech envelope that represents slow amplitude modulations carrying prosodic and syllabic cues^4,5^. Electrophysiological studies consistently show that brain activity in the delta (1–4 Hz) and theta (4–8 Hz) bands track the speech envelope^2,6–12^, putatively facilitating the decoding of acoustic, linguistic, and higher-order information from continuous speech^13,14^. Notably, cortical speech tracking is not purely a bottom-up response. It has been shown to be modulated by top-down cognitive factors such as attention^15,16^, working-memory load^17^, listening effort^18^, and prior expectations^19^. Cortical speech tracking is now widely recognised as an important mechanism for auditory processing, yet the precise neural and computational processes that give rise to it remain to be elucidated.

Tracking of speech rhythm appears to extend beyond brain rhythms, manifesting also in other bodily rhythms. Recent work shows that eye movements, blinks, and pupil dynamics align with speech features in synthesised speech^20^, short sentences^21^, and naturalistic continuous speech^22–24^, with a consistent link between this tracking and attentional mechanisms. Specifically, pupil response stands out as a temporally rich signal in recent oscillation-based frameworks^25–27^, governed by autonomic nervous system activity, with sympathetic and parasympathetic influences driving pupil dilation and constriction respectively^28^. Ongoing fluctuations in pupil dilation reflect arousal, stemming from subcortical neuromodulatory systems^29^, primarily the locus coeruleus-norepinephrine pathway, which is often characterised by phasic (i.e. event-related response) and tonic (i.e. task-free, spontaneous fluctuations) modes^27,30^. Furthermore, pupil dynamics covary with the power in several cortical frequency bands^31^. On the other hand, eye-movement based speech tracking has been linked to cortical tracking of speech, implying motor and attentional engagement in auditory processing^21^^,^^24^. Together, these findings suggest that eye responses support auditory speech perception and may serve as a proxy for sensorimotor engagement. Still, further research is needed to establish the reliability and generalisability of these measures and to examine top-down influences beyond attention, as well as their potential interactions, in ocular speech tracking.

One such influence may be the emotional content of speech, which is particularly relevant as emotions are inherent at nearly every level of spoken communication, from phonetic detail to semantic, grammatical, and discourse cues^32^. Research on emotional speech processing has mostly focused on prosody, that is, the suprasegmental modulation of tone, stress, and rhythm, which serves both linguistic and emotional functions^33,34^. On the other hand, speech tracking research has largely focused on the linguistic functions of prosody, neglecting its social and emotional dimensions (see^35,36^:. Yet linguistic and emotional functions of prosody are closely intertwined with shared acoustic parameters and neural pathways^34,37,38^,, making consideration of the emotional aspects equally essential. Indeed, it has already been suggested that connecting patterns of envelope tracking to the perception of emotions could offer practical insights for clinical populations characterised by prosodic difficulties, as well as for typically developing individuals^33^. Here, we empirically test the relationship between the emotion content in continuous speech and ocular responses.

Emotions are known to have preferential access to attentional resources, thereby facilitating adaptive behaviours in complex environments^39–43^. Behavioural studies consistently report enhanced performance in a variety of tasks, such as faster reaction times and greater accuracy when processing emotional speech compared to neutral speech^44–48^. Neuroimaging work further reveals distinct electrophysiological and hemodynamic patterns for emotional prosody^49–53^, suggesting emotion may impact early auditory processing. Taken together, these findings make emotions a strong candidate for modulating speech tracking at both neural and physiological levels.

The present study was guided by two complementary aims. First, we sought to extend existing evidence for speech rhythm tracking by ocular responses during passive listening to continuous speech, focusing specifically on pupil dilation and eye movements. Second, we tested whether and how emotional factors relate to ocular speech tracking. To address these aims, we conducted two studies: an initial validation study assessing the consistency of emotional responses elicited by our naturalistic speech stimuli across a large sample (*N* = 100), followed by the main experiment (preregistration: https://doi.org/10.17605/OSF.IO/X2SRW), in which participants (*N* = 41) listened to the same stimuli while we recorded electrooculography and pupillometry. We then quantified the coupling between the speech signal and each ocular measure and examined its relationship to subjective ratings of emotional arousal and valence, listeners’ mood and empathy. We tested the hypothesis that higher emotional arousal would enhance ocular speech tracking, potentially through increased attention. We did not make a priori hypothesis about the effects of valence and its potential interactions with arousal since less is known about how these factors shape speech processing in naturalistic settings.

### Study 1: Validating emotional speech stimuli

In Study 1, we established a set of naturalistic speech stimuli for use in Study 2 and assessed the interrater reliability of the emotional responses they evoked. Although several validated emotional speech databases are available, existing resources were unsuitable for our aims due to limitations including speaker accent, stimulus length, reduced naturalness of delivery, and copyright restrictions. We therefore selected two TED talks (Talk 1: 13 min; Talk 2: 12 min), each varying in emotional content over the course of the talk, and asked participants to provide intermittent ratings of arousal and valence. Our central question was whether listeners would show reliable emotional responses to these naturalistic excerpts. A complementary analysis of the acoustic features predicting these ratings is reported in Supplementary Materials.

The treatment of participants adhered to the principles of the Declaration of Helsinki. Written informed consent was obtained from all participants. The study was approved by the School of Social Sciences Ethics Committee at the University of Dundee (Approval No: UoD-SHSL-PSY-PG-2023-164). Participants received compensation in the form of monetary payment (£5) or course credit, depending on their preference.

## Materials and methods

### Participants

A sample of *N* = 100 volunteers (77 female, 20 male, and 3 non-binary participants; *M*_age_= 24.5 years, age range = 17–48 years, *SD*_*age*_= 6.84) were recruited via the department’s participant pool and advertisements. All participants reported advanced English proficiency and no prior diagnoses of speech or language disorders, neurodevelopmental conditions, or psychological or neurological disorders. Participants completed the *Quick Hearing Test*(QHT^54^; to assess their self-reported hearing ability. Four participants obtained scores (28,28,32,38 out of 60) that may suggest slightly diminished hearing (hearing test is recommended for scores ≥ 20). Participants completed additional self-report measures, including the *Brief Mood Introspection Scale*(BMIS^55^, which assesses current mood, and the *Toronto Empathy Questionnaire*(TEQ^56^, measuring emotional understanding and responsiveness to others. Participants’ average mood score was *M* = 49.2 (*SD* = 5.78) on the valence dimension and *M* = 27.8 (*SD* = 3.75) on the arousal dimension, while the average total empathy score was *M* = 50.8 (*SD* = 6.50).

### Speech stimuli

We used two audio recordings of TED Talks delivered by female speakers with British accents (Talk 1: “You can be fat and happy” by Sofie Hagen; Talk 2: “Breaking out of concrete: six ways out of depression” by Bethany Rose). We edited the original recordings to shorten long silences, audience reactions, such as laughter and applause, and segmented each talk into speech chunks ranging from 7.27 s to 22.35 s (*M* = 13.90, *SD* = 3.81 s). We ensured that speech chunks were emotionally coherent. This process resulted in 101 distinct speech chunks (N_talk1_ = 55, N_talk2_ = 46), each containing between one and six sentences of varying structure.

We presented all speech stimuli using PsychToolbox 3.0.17^57^, delivering audio playback with high-quality wired headphones (Sennheiser HD-25, 70 Ω). Additionally, we amplitude-normalised the stimuli to maintain consistent volume levels across all excerpts. Audio files had a sampling rate of 44,100 Hz. The speech stimuli are publicly available on the OSF server (https://osf.io/dsvzh/files/osfstorage).

### Procedure and task

Participants were tested in a quiet room while seated at an approximate distance of 65 cm from the screen. They were instructed to fixate on the circle presented at the centre of the screen, to listen attentively to the speech stimuli, and to rate each chunk on arousal and valence scales. To familiarise themselves with the task, they first completed a practice trial in which they listened to three speech chunks from a TED Talk that was not included in the main task. They were allowed to adjust the volume to their preferred level before the main task.

The main task consisted of two blocks, each corresponding to one of the TED Talks. The order of the blocks was randomised, whereas the order of the speech chunks was kept constant to preserve the natural flow of the narrative. A self-paced break was provided between the two blocks. Participants rated their own felt emotions on visual analogue scales for valence and arousal separately. For valence ratings, participants answered the question “How did the last speech chunk make you feel?” using a visual analogue scale from −100 to 100, anchored by “Very negative” and “Very positive”. Arousal was measured with the question “How intense was your emotional response to the speech chunk?”, accompanied by a visual analogue scale ranging from 0 to 100 with anchors “Low arousal” and “High arousal”. Participants indicated their response by placing a vertical line on the scale through a mouse click. At the end of each talk, participants completed a familiarity rating to assess whether they had encountered the speech material before. The session, including instructions, questionnaires, and two passive listening blocks, lasted approximately one hour.

### Emotion model

Although there is not yet consensus in the literature on how emotions should be modelled^58^, we here adopted a well-established dimensional approach to conceptualise emotions. In the dimensional approach, an emotion can be represented by a specific point within a continuum defined by a small set of underlying dimensions, which may be more appropriate for a more nuanced assessment of emotional experience compared to discrete models^59,60^. Specifically, we used the circumplex model of affect, which postulates that emotions can be mapped into two orthogonal dimensions: valence, referring to the degree of pleasantness of an emotion, and arousal, reflecting its level of intensity or activation^61^.

### Interrater reliability analysis

To quantify the reliability of emotion ratings across participants, we calculated intraclass correlation coefficients (ICCs) separately for arousal and valence. Specifically, we used the ICC(2,k) model, a two-way random-effects model that estimates the consistency of average ratings across multiple raters sampled from a larger population^62^. This model is appropriate when the same set of raters evaluate all items and the goal is to generalise to other raters. ICC values were interpreted according to the thresholds proposed by^62^: poor (<.50), moderate (.50–.75), good (.75–.90), and excellent (>.90).

## Results

### Interrater reliability

Intraclass correlation coefficients indicated that both emotion dimensions were rated with substantial consistency across participants. For the average rating across participants, valence showed excellent interrater reliability, ICC(2,k) =.99, 95% CI [.99,.99], *F*(100, 9900) = 98.0, *p* <.001, while arousal showed good reliability, ICC(2,k) =.87, 95% CI [.83,.90], *F*(100, 9900) = 9.4, *p* <.001.

Descriptively, chunk-level mean ratings spanned a wide emotional range across both talks, confirming that the excerpts elicited varied affective responses. For Talk 1, mean valence ratings ranged from −65.3 to 56.9 (*M* = −10.5, *SD* = 35.7), and mean arousal ratings ranged from 45.7 to 72.1 (*M* = 59.5, *SD* = 6.2). For Talk 2 (46 chunks), mean valence ranged from −69.9 to 58.9 (*M* = 9.9, *SD* = 38.5), and mean arousal ranged from 47.6 to 73.2 (*M* = 58.4, *SD* = 7.0). The distributions of arousal and valence ratings across both talks are provided in the Supplementary Materials (Figure S1), illustrating the temporal variation in emotion ratings of each chunk.

## Discussion

Study 1 showed that naturalistic excerpts from TED talks reliably evoke distinct emotional responses across listeners. Interrater agreement was excellent for valence and good for arousal, and chunk-level ratings spanned a wide range of valence and a moderate range of arousal dimensions. This indicates that the stimulus set captures meaningful emotional variability, rather than clustering around neutral values. The higher agreement for valence than arousal likely reflects that valence judgements can be extracted from more overt cues, such as lexical content and affective prosody, whereas arousal judgements may depend more strongly on subjective perceptual processes.

Together, these findings establish the stimuli as a suitable basis for Study 2, in which we examined how subjective emotion ratings modulate ocular tracking of the speech envelope.

### Study 2: Ocular responses to speech rhythm: Investigating the effects of perceived emotions

In Study 2, we aimed to examine how ocular physiological signals (pupil dilation, horizontal and vertical EOG) interact with the quasi-rhythmic structure of continuous speech and how emotions shape this tracking. Recent findings indicate that, in addition to the well-established alignment between speech acoustics and cortical rhythms, ocular dynamics may also track speech^20–24^. However, available evidence is currently insufficient and inconsistent to determine whether speech tracking generalises to different ocular signals. Moreover, it is unlikely that such tracking operates independently of stimulus characteristics, listening environments, as well as cognitive and affective influences. While attention has been shown to modulate the temporal alignment between speech and pupil dilation^20^, as well as eye movements^21^^,^^24^, it is unclear how other factors may influence ocular speech tracking. Here, we investigated how the perceived emotional characteristics of the speech material and other emotion-related factors (listener’s own mood state and trait empathy) were linked to ocular speech tracking.

There are (at least) four reasons to expect that emotions may shape speech tracking in ocular responses. First, emotional expressive speech shows more pronounced and frequent acoustic variability than emotionally neutral speech^63,64^. Such emotion-induced changes in the speech signal are likely to influence envelope tracking. Second, there is robust evidence that emotionally expressive speech in different forms (isolated words or short sentences) is processed differently from neutral speech at both neural and behavioural levels^37,44,65^. Third, emotional stimuli are speculated to be prioritised in attention^42^, implying that emotion-related changes in attentional mechanisms could shape the extent to which ocular signals align with the speech signal. Lastly, the ocular measures we used have been associated with auditory emotional processes. Specifically, emotionally arousing stimuli have been shown to elicit greater pupillary response, potentially through increased autonomic nervous system activity^66–70^. Beyond basic autonomic responses to arousing stimuli, some evidence suggests that pupil dilation is sensitive to emotional valence^68,71,72^ and emotional authenticity^73^, and is further associated with audiovisual integration^74^ as well as emotion-related decision processes^75^ in response to vocal emotional stimuli (see^76^ for an overview of pupillometry studies in the auditory domain). Direct evidence for the role of eye movements in the vocal emotion literature is scarce, with most support coming from cross-modal studies showing that emotional prosody can facilitate gaze behaviour in visual tasks^77–79^. Likewise, only a limited number of studies have examined eye movements with a naturalistic speech listening paradigm, and those that have done so have focused on listening effort^80^^,^^81^. Although eye movement and pupil dynamics have been examined within speech-rhythm frameworks, their sensitivity to emotional dimensions has not been evaluated. To our knowledge, the present study is the first to test whether emotional dimensions of speech and listeners’ mood states modulate speech tracking as reflected in ocular dynamics under naturalistic listening conditions.

The main analyses were preregistered prior to data analysis (https://doi.org/10.17605/OSF.IO/X2SRW). However, there were some small deviations from the preregistration as we encountered new analytic developments, which are described below where relevant.

## Materials and methods

### Participants

A total of N = 44 participants completed the experiment. Data from three participants were excluded for the following reasons: one withdrew from the study, one reported intentionally providing neutral ratings for every chunk, and for one, technical issues resulted in a failure to save electrooculographic data. The final sample consisted of 41 participants (28 female, 13 male; *M*_age_ = 23.2 years, age range = 17–39 years, *SD*_*age*_= 5.56; 36 right-handed, 3 left-handed, 2 cross-dominant). Thirty-nine participants provided complete datasets; for the remaining two, only partial data were analysed: one contributed only EOG data due to missing pupil data, and the other completed only one talk.

All participants were native English speakers. They reported no prior diagnosis of speech or language disorders, neurodevelopmental conditions, or psychological or neurological disorders. Self-report measures were identical to Study 1, including the *Quick Hearing Test* (*QHT*^54^), *Brief Mood Introspection Scale* (*BMIS*^55^), and *Toronto Empathy Questionnaire* (*TEQ*^56^). 39 participants had normal hearing, while two obtained scores (23 and 24 out of 60) that may suggest slightly diminished hearing (hearing test is recommended for scores ≥ 20). Participants’ average mood score on the valence dimension was *M* = 48.8 (*SD* = 5.71), the average mood score on the arousal dimension was *M* = 26.4 (*SD* = 3.69), and average empathy score was *M* = 50.2 (*SD* = 5.85).

All aspects of the study adhered to the principles of the Declaration of Helsinki. Written informed consent was obtained from all participants. The study was approved by the School of Social Sciences Ethics Committee at the University of Dundee (Approval No: UoD-SHSL-PSY-PG-2023-164). Participants received compensation in the form of monetary payment (£15) or course credit, depending on their preference.

### Stimuli and task

**Fig. 1:**
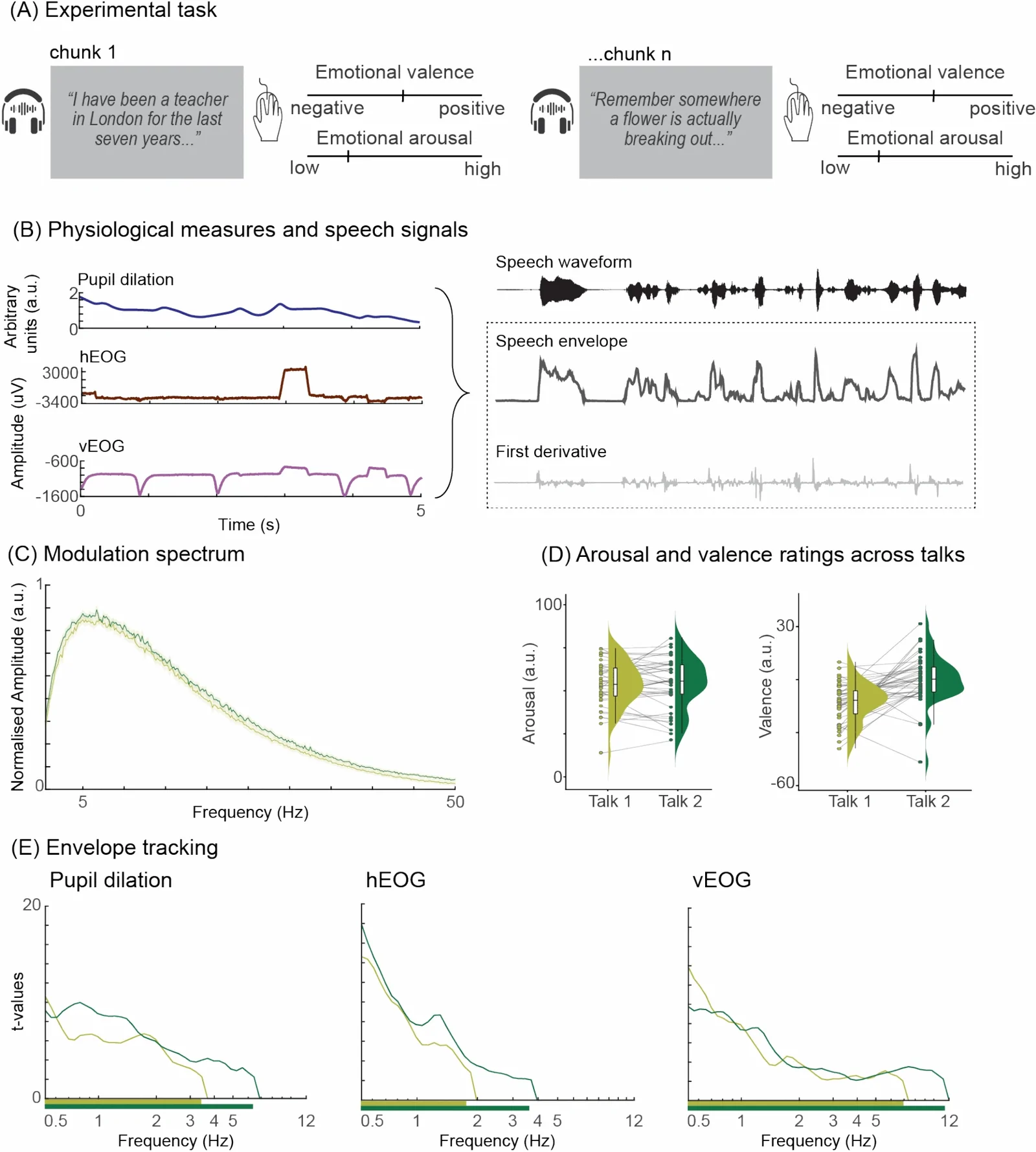
Overview of the experimental task and measures. (**A**) A passive listening and rating task was used in Study 1. Participant listened to speech chunks (between 7 and 22 s) while fixating on a circle with the same luminance as the background. Then they rated each chunk on arousal and valence ratings. (**B**) In Study 2, participants completed the same passive listening and rating task while pupillometry and electrooculogram signals were recorded simultaneously (example signals in left panel). We then examined how each eye signal tracked the acoustic speech signal, that is, the speech envelope and its first derivative extracted from the waveform (example signals in right panel). (**C**) Modulation spectrum of both talks. Light and dark green lines indicate average values across 6-s segments for Talk 1 and Talk 2 respectively, with shaded areas representing standard error of the mean. (**D**) Raincloud plots showing mean arousal and valence ratings across speech chunks for Talk 1 and Talk 2 (*N* = 41). Each point represents an individual participant’s mean rating; half-violin plots illustrate the kernel density of these means. Light grey lines connect each participant’s mean emotion ratings between talks, emphasising within-subject changes. (E) All eye signals track the acoustic speech signal at slow frequencies. Panels show significant speech tracking for pupil, horizontal EOG (hEOG), and vertical EOG (vEOG) from left to right. Talk 1 is represented with a light green line and Talk 2 with a dark green line. The y-axis is logarithmically scaled to emphasise differences across lower frequencies. t-values are derived from comparing z-scored mutual information (MI) values against zero. Light and dark green bars below the x-axis indicate frequency ranges where cluster-based permutation tests revealed significant effects for Talk 1 and Talk 2, respectively.

We used the same two TED talks as speech stimuli as in the first study. Speech rate was computed in Praat (Version 6.0.18; Boerma & Weenink, 2007), with 3.50 Hz for the first talk and 3.22 Hz for the second talk^82^. The procedure and task were also identical to that in Study 1. However, in Study 2, we recorded eye movements and pupil size throughout the experiment using electrooculography (EOG) and pupillometry, respectively (Fig. 1A). The session, including setup, instructions, questionnaires, and two passive listening blocks, lasted approximately 1.5 hours.

### EOG data acquisition and preprocessing

EOG data was acquired using a BioSemi ActiveTwo system with a sampling rate of 512 Hz. We used EOG to quantify eye movements, rather than eye-tracker–based gaze measures, because EOG signals are straightforward to obtain without additional calibration demands and provide a meaningful representation of blink-related activity that contributes to the continuous signal. Four Ag/AgCl electrodes were used to capture horizontal and vertical eye movements: two electrodes were placed at the outer canthi of both eyes to measure horizontal EOG (hEOG), and two were placed above and below the left eye to measure vertical EOG (vEOG). A scalp electrode placed at the vertex served as the reference. Electrode signals were visually inspected during recording to ensure data quality. The absolute offset value was kept below 20 μV and never exceeded 30 μV.

EOG data were pre-processed using custom MATLAB scripts (MATLAB 2023a, The MathWorks Inc.) with the FieldTrip toolbox^83^. Vertical EOG was calculated as the difference between electrodes above and below the left eye, while horizontal EOG was calculated as the difference between electrodes placed at the left and right temples. The continuous EOG recordings were segmented into epochs corresponding to the exact duration of each speech chunk. The segmented EOG signals were then bandpass filtered between 1 Hz and 8 Hz using a fourth-order Butterworth filter (applied forward and reverse) and downsampled to 250 Hz.

To address the potential contribution of blinks to vEOG-speech coupling as an additional control analysis, blinks were identified and removed from the vEOG signal via interpolation. Details of the blink-removal process and the corresponding mutual information analysis are reported in Supplementary Materials.

### Pupil data acquisition and preprocessing

The pupil signal was recorded using an EyeLink II head-mounted eye-tracking system (SR Research, Osgoode, ON, Canada) for the first cohort of participants (*N* = 23). As several participants reported discomfort with the headset, the remaining participants (*N* = 24) were tested using a desktop-mounted EyeLink 1000 Plus system (SR Research Ltd., Mississauga, ON, Canada) equipped with a chin rest (SR Research Head Support) to stabilise head position. In both setups, pupil signal was continuously recorded from the right eye at a sampling rate of 250 Hz. Before each talk, a standard five-point calibration and validation procedure was performed to ensure accurate pupil measurements. The recording room was dimly lit, and both the fixation circle and background were kept at equal luminance levels to minimise luminance-driven changes in pupil diameter. We conducted a control analysis to assess whether eye-tracking device type influenced pupil–speech mutual information values. Including device type as an additional fixed effect neither improved model fit nor was it a significant predictor of pupil–speech MI values (*χ*^2^(1) = 1.20, *p* =.27). The patterns of emotion-related effects remained robust, except that the main effect of listeners’ mood arousal was no longer statistically significant after accounting for device type. Overall, the results of the control analysis indicate that the observed findings were not driven by device-related variability (see Supplementary Materials, Table S2).

Pupil data were pre-processed using custom MATLAB scripts (available at https://github.com/anne-urai/pupil_preprocessing_tutorial; as used in^31,84^ in combination with functions from the FieldTrip toolbox^83^. Pupil area values were converted to pupil diameter. Blinks were automatically detected and linearly interpolated, and small eye-tracker artifacts such as spikes and jumps were identified and corrected using the same procedure. We deviated from the preregistration by interpolating blink-related activity in the pupil signal rather than excluding blink windows, as the analysis pipeline required a continuous signal without missing data. Following blink interpolation, the pupil data were band-pass filtered between 1 and 20 Hz using a second-order zero-phase Butterworth filter (applied forward and reverse) to remove slow drifts. To ensure precise temporal alignment between eye-movement and pupil signals, a cross-correlation between z-scored pupil and vertical EOG signals was computed to identify temporal offset. The identified peaks were visually inspected for accuracy. Finally, the pupil and eye movement data were segmented into trials corresponding to the stimulus chunks.

### Speech spectral characteristics and envelope extraction

To compare the spectral characteristics of the two talks, we computed the modulation spectrum for both talks by segmenting speech excerpts into 6-s chunks^25,26,85^. Modulation spectra were highly similar, including the 0–12 Hz frequency range relevant to our analysis (Fig. 1B). We extracted the wideband speech envelope and its first derivative for each speech chunk. The speech waveform was filtered into eight frequency bands ranging from 100 to 8000 Hz using third-order, forward and reverse Butterworth filters. These bands were spaced equidistantly according to the cochlear frequency scale^86^. As all recordings were in stereo format, extraction was performed separately for each channel. Within each channel, the filtered signals for each frequency band were Hilbert-transformed, and the magnitude of the signal was extracted to compute the envelope. The resulting envelopes across all eight frequency bands were then averaged for each channel. Finally, the envelopes from both channels were averaged to generate a single wideband envelope per speech chunk. Last, the speech signals were downsampled to 250 Hz.

### Mutual information analysis

We quantified the correspondence between continuous eye and speech signals using a mutual information (MI) framework^87–89^. This approach robustly captures non-linear dependencies between time series using a Gaussian copula-based instead of a binning approach^88^.

MI (in bits) between the eye signals (continuous pupil dilation, hEOG, and vEOG) and the speech signals (envelope and its derivative) was computed with all signals transformed in the frequency domain (using a continuous wavelet transformation for 46 logarithmically spaced frequencies between 0.5 and 12 Hz)^87^. MI values were estimated for 39 lags between 100 ms and 2400 ms (in 60 ms steps), as it is not possible to estimate a single optimal lag per individual for pupil dilation and eye movements. This is because eye activity reflects several cognitive (speech-related and emotion-related) processes, which all follow different temporal dynamics (e.g^23^). MI values were computed separately for each participant, speech chunk, and eye signal.

Although our preregistration proposed examining MI across frequency bands linked to linguistic units (syllables, words, phrases, sentences), we anchored the analyses to the speech envelope and its first derivative, which offer a more assumption-free representation of speech dynamics.

### Statistical analysis

We first tested whether ocular speech tracking (for pupil dilation, vertical and horizontal EOG) was present when compared against chance-level (i.e., whether MI values for speech-eye correspondence were above chance level). To this end, observed MI values were first normalised using surrogate MI values. This surrogate data was created by randomly shuffling 3-second segments of the speech envelope and derivative^25,26^. This way, any temporal correspondence between the speech and eye signals was removed. Then, we estimated surrogate MI values for shuffled envelope signals and eye signals. In total, we computed 100 surrogate MI values for each participant, speech chunk, eye signal, and frequency. This was done for 5 equally spaced lags between 100 and 2260 ms, covering the full temporal range of lags. We did not compute surrogate data for all 39 lags as this would have been too computationally intensive. MI values across the five chosen lags were averaged, and surrogate MI was used to z-score observed MI values, which was then compared against zero using a dependent-samples t-tests for all frequencies^87^. We used a cluster-based permutation test with a Monte Carlo randomisation procedure (1000 permutations) to correct for multiple comparisons across frequencies (Maris & Oostenveld, 2007). Clusters were identified along the frequency dimension using a cluster-defining threshold of *α* = 0.05 (as well as a cluster-level alpha of *α* = 0.05), and significance was determined based on the 95th percentile of the cluster-level null distribution, controlling the family-wise error rate.

To assess the relationship between emotion-related factors and ocular speech tracking, we fitted Linear Mixed-Effects Models (LME) separately for pupil, hEOG, and vEOG in R (v4.4.2^90^; using the *lme4*^91^. The outcome variable was the mutual information between the speech and the corresponding eye signal. Fixed effects were emotion ratings (speech arousal and speech valence) and participants’ mood scores (mood arousal and mood valence). We included the interaction between arousal and valence to test whether their combined influence on ocular tracking differed from their main effects. To test whether the relationship between speech emotions and ocular tracking depended on participants’ current mood state, we also included the interaction between emotion ratings and mood scores. We further entered chunk order (to account for changes in tracking over time), participant’s empathy scores, and acoustic predictors (pitch, HNR, F_1_, see Supplementary Materials) into the models as fixed factors. All continuous predictors were z-scored prior to model fitting. Talk was included as a categorical predictor and coded using treatment contrasts, with Talk 1 as the reference level (i.e., Talk 1 was coded as 1, and Talk 2 was coded as 2). Following the maximal random effects approach^92^, we included random intercepts for participants, speech chunks, and speech-eye lags to account for dependencies introduced by the repeated-measures design. Speech-eye lags were also included as random factor to get an initial overview of effects that were related to speech tracking. We initially considered more complex random-effects structures, including by-participant random slopes for arousal and valence. However, these models did not converge reliably and produced singular fits. We therefore retained a random-intercepts-only structure. The final model, hereafter referred to as the *full model*, was:

$$Speech\: Tracking \sim Speech\: Arousal* Speech\: Valence\: +$$

$$Speech\: Arousal\: *\: \left(Mood\: Arousal\: +\: Mood\: Valence\right)+$$

$$Speech\: Valence\: *\: \left(Mood\: Arousal\: +\: Mood\: Valence\right)\:+$$

$$Talk\: ID\: +\: Time\: +\: Empathy\: +\: Pitch\: +\: HNR \:+\:F1\:+$$

$$\left(1\: \right|Participant \:ID) \:+ \:(1\: | Chunk\: ID) + (1\: | Lag)$$

To estimate the temporal evolution of effects across the range of lags, we fitted separate models for each individual speech-eye lag. This was identical to the model structure above, except that lag was not included as random factor. The resulting *t*-statistic curves are shown in Supplementary Materials (Figure S2).

Overall model fit was evaluated by comparing the full model to a null model containing only random intercepts. For each fixed effect, p-values were obtained using Satterthwaite’s approximation for degrees of freedom (*lmerTest*^93^;. To control for multiple comparisons across predictors, false discovery rate (FDR) correction was applied to the resulting p-values within each model^94^. This correction was performed separately for each dependent measure. Diagnostic plots did not reveal any obvious deviations from the assumptions of multilevel analysis (linearity, homogeneity of variance, multicollinearity, and normality of residuals). Models were estimated using restricted maximum likelihood for model comparison purposes, and the significance level was set at *α* = 0.05.

Note that we initially included both linear and quadratic valence terms in the models to capture potential nonlinear effects of valence. These models revealed several main and interaction effects related to quadratic valence. However, the inclusion of the quadratic term introduced complex interaction patterns, obscuring a clear interpretation. For clarity and parsimony, we opted to report the model with only linear component of valence in the main text. Full results from the quadratic models are provided in the Supplementary Materials.

## Results

### Ocular tracking of speech rhythm

We first examined whether participants’ ocular signals tracked the acoustic speech envelope and its first derivative significantly above chance levels using dependent-samples t-tests with cluster-based permutation to control for multiple comparisons across frequencies (see Fig. 1D). Real and surrogate MI values of each ocular signal are presented in Supplementary Materials. For pupil dilation, we observed a significant positive cluster for the first talk between 0.53 Hz and 3.45 Hz (cluster *t*_sum_ = 164.34, Cohen’s d_Peak_ = 3.31, *p* =.001) and for the second talk between 0.53 Hz and 6.43 Hz (cluster *t*_sum_ = 233.62, Cohen’s d_Peak_ = 3.12, *p* =.001). For hEOG, a positive cluster emerged between 0.53 Hz and 1.85 Hz (cluster *t*_sum_ = 152.05, Cohen’s d_Peak_ = 4.57, *p* =.001) for the first talk and between 0.53 and 3.69 Hz (cluster *t*_sum_ = 206.56, Cohen’s d_Peak_ = 5.60, *p* =.001) for the second talk. For vEOG, we found a significant positive cluster between 0.53 Hz and 6.89 Hz (cluster *t*_sum_ = 194.12, Cohen’s d_Peak_ = 4.34, *p* =.001) for the first talk, and between 0.53 Hz and 11.20 Hz (cluster *t*_sum_ = 218.02, Cohen’s d_Peak_ = 3.05, *p* =.001) for the second talk. Notably, this effect persisted in the blink-cleaned vEOG signal (see Supplementary Materials, Figure S4). These findings suggest that all eye signals systematically tracked slow fluctuations in the acoustic speech signal.

### Predictors of pupil speech tracking

We tested the relationship between pupil speech tracking and our predictors (speech arousal, speech valence, mood arousal, mood valence, trait empathy, pitch, HNR, F_1,_ speech chunk order, and talk ID) with a linear mixed-effects model. The full model included all used speech-eye lags as random factors to assess the overall role of predictors. Additionally, we computed single-lag models to assess the temporal evolution of each predictor (see Supplementary Materials Figure S2).

We observed a positive main effect of *speech arousal* (*β* = 0.02, *p*_FDR_ <.001) in the main model, suggesting stronger pupil tracking for high-arousal speech than low-arousal speech. Single-lag models revealed peak effects of arousal at 1780 ms. We observed significant main effects of both trait empathy (*β* = −0.03, *p*_FDR_ =.04) and mood arousal (*β* = 0.04, *p*_FDR_ =.03), with lower trait empathy and high-arousal mood associated with stronger pupil tracking of speech. We also observed a significant main effect of HNR (*β* = −0.20, *p*_FDR_ =.023), with higher HNR, indicative of a cleaner and more tonal voice quality, associated with weaker speech tracking. No significant main effects were observed for the remaining predictors (see Table 1).

**Table 1:** Summary table for main model predicting pupil-speech MI values. LL = Lower limit, UL = Upper limit, A = Arousal dimension, V = Valence dimension. Fixed-effect estimates (β) are reported with confidence intervals (95% CI) computed using the Wald method. *p* values were false discovery rate (FDR) corrected. Significant predictors are highlighted in bold. The model included random intercepts for participants, speech chunks, and speech-eye lags. *p <.05, **p <.001.

Predictors	Estimates	95% CI		*p*_*uncorrected*_	*p*_FDR_
		LL	UL		
Intercept	0.17	−0.05	0.38	.127	.170
**Arousal**	0.02	0.01	0.02	<.001**	<.001**
Valence	−0.01	−0.01	−0.001	.033*	.061
**Mood (A)**	0.04	0.01	0.07	.013*	.034*
Mood (V)	0.01	−0.02	0.04	.405	.498
Time-in-talk	0.03	−0.08	0.14	.566	.566
**Trait empathy**	−0.03	−0.06	−0.01	.020*	.046*
Pitch	0.03	−0.13	0.18	.730	.730
F_1_	0.06	−0.09	0.21	.454	.519
HNR	−0.20	−0.35	−0.06	.007*	.023*
Talk	−0.37	−0.78	0.03	.075	.120
**Arousal × Valence**	0.02	0.02	0.03	<.001**	<.001**
**Arousal × Mood (A)**	0.015	0.01	0.02	<.001**	<.001**
Arousal × Mood (V)	−0.004	−0.01	0.001	.089	.129
**Valence × Mood (A)**	−0.01	−0.02	−0.01	<.001**	<.001**
Valence × Mood (V)	−0.005	−0.01	−0.000	.034*	.061

**Fig. 2:**
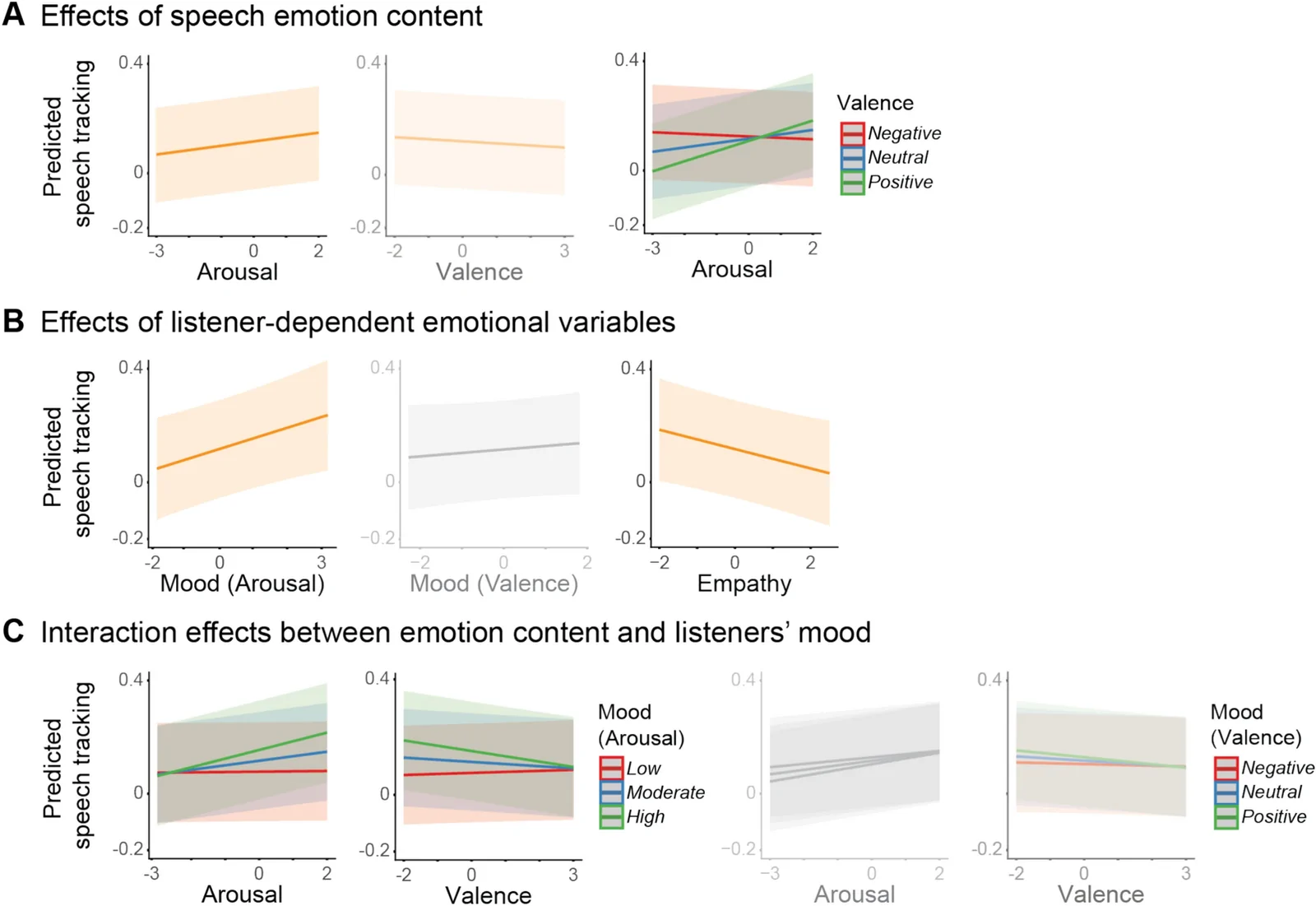
Relationship between pupil tracking of speech and emotion-related predictors. Each plot displays the predicted MI values from the model (y-axis) as a function of the corresponding predictor (x-axis), with shaded areas representing 95% confidence intervals. Variables that did not predict pupil envelope tracking are shown in gray; predictors that did not survive FDR correction are shown in paler colours. (**A**) Effects of subjective emotion ratings of speech on pupil speech tracking. The model revealed significant main effects of arousal on pupil tracking of speech signal. The main effect of valence did not remain significant after correction for multiple comparisons; however, its interaction with speech arousal predicted pupil tracking of speech. (**B**) Effects of listener-dependent emotional variables (arousal and valence dimensions of current mood and trait empathy) on pupil speech tracking. High-arousal mood was associated with overall stronger speech tracking, whereas the main effect of mood on valence dimension did not predict speech tracking. Lower trait empathy predicted stronger speech tracking. (**C**) Interactions between subjective emotion ratings of speech and listener-dependent emotional variables (arousal and valence dimensions of current mood) and their effect on pupil speech tracking. The arousal dimension of mood significantly modulated the effects of both speech arousal and speech valence on pupil tracking. In contrast, interactions involving mood valence either did not survive FDR correction or were not significant predictors. Overall, pupil tracking of speech signals was influenced by both stimulus-driven and listener-dependent emotional factors.

The full model also revealed several interaction effects. First, we found a two-way interaction effect of *speech arousal* × *speech valence* (*β* = 0.02, *p*_FDR_ <.001; peak effect observed at 880 ms in the single-lag models). This suggests that when speech was positive in valence, high-arousal speech was tracked more strongly than low-arousal speech. In contrast, when speech was negative in valence, this relationship reversed, with low-arousal speech being tracked more strongly (see Fig. 2A).

Second, we found interaction effects between speech emotion (i.e. arousal or valence ratings) and individuals’ own mood in both arousal and valence dimensions (see Fig. 2C*)*. When participants were in a high-arousal mood, they tracked high-arousal speech stronger, while this relationship was absent for people in a low-arousal mood (*arousal × mood arousal*: *β* = 0.015, *p*_FDR_ <.001; peak effect observed at 2020 ms in the single-lag models). Additionally, individuals in a high-arousal mood tracked negative speech more strongly than positive speech, whereas those in a low-arousal mood tended to track positive speech more strongly (*speech valence* × *mood arousal*: *β* = −0.01, *p*_FDR_ <.001; peak effect observed at 2260 ms in the single-lag models). Regarding *mood valence*, while we did not observe a significant effect for its interaction with speech arousal; *speech valence* × *mood valence* predicted pupil speech tracking. Yet, this effect did not survive correction for multiple comparisons (*p*_uncorrected_ =.03; *p*_FDR_ =.06)

### Predictors of hEOG speech tracking

The full model with hEOG-speech tracking as outcome variable revealed that only the main effect of *arousal* was significant (*β* = −0.01, *p*_FDR_ =.036; peak effect observed at 1600 ms in the single-lag models), suggesting that horizontal eye movements tracked low-arousal speech stronger than high-arousal speech. *Mood arousal* (*p*_uncorrected_ =.04; *p*_FDR_ =.10) and HNR (*p*_uncorrected_ =.03; *p*_FDR_ =.08) also predicted hEOG-speech coupling, however their effect did not survive after FDR correction. None of the remaining predictors showed a significant main effect (see Table 2).

**Table 2:** Summary table for the mixed effects model predicting hEOG-speech MI values. A = Arousal dimension, V = Valence dimension. Fixed-effect estimates (β) are reported with confidence intervals (95% CI) computed using the Wald method. *p* values were false discovery rate (FDR) corrected. Significant predictors are highlighted in bold. The model included random intercepts for participants speech chunks and speech-eye lags. *p <.05 **p<.001.

Predictors	Estimates	95% CI		*p*_*uncorrected*_	*p*_FDR_
		LL	UL		
Intercept	0.18	−0.04	0.40	.117	.187
**Arousal**	−0.01	−0.01	−0.002	.011*	.036*
Valence	−0.001	−0.01	0.01	.692	.704
Mood (A)	0.03	0.01	0.05	.045*	.102
Mood (V)	0.02	−0.01	0.04	.192	.257
Time-in-talk	0.001	−0.09	0.14	.667	.704
Trait empathy	−0.01	−0.03	0.02	.506	.622
Pitch	−0.01	−0.14	0.21	.704	.704
F_1_	0.11	−0.05	0.26	.184	.257
HNR	−0.17	−0.32	−0.01	.030*	.081
Talk	−0.02	−0.82	0.02	.065	.130
**Arousal × Valence**	0.01	0.01	0.01	<.001**	<.001**
**Arousal × Mood (A)**	0.02	0.01	0.02	<.001**	<.001**
**Arousal × Mood (V)**	−0.01	−0.02	−0.01	<.001**	<.001**
Valence × Mood (A)	−0.004	−0.01	0.001	.087	.155
**Valence × Mood (V)**	−0.01	−0.01	−0.01	<.001**	<.001**

**Fig. 3:**
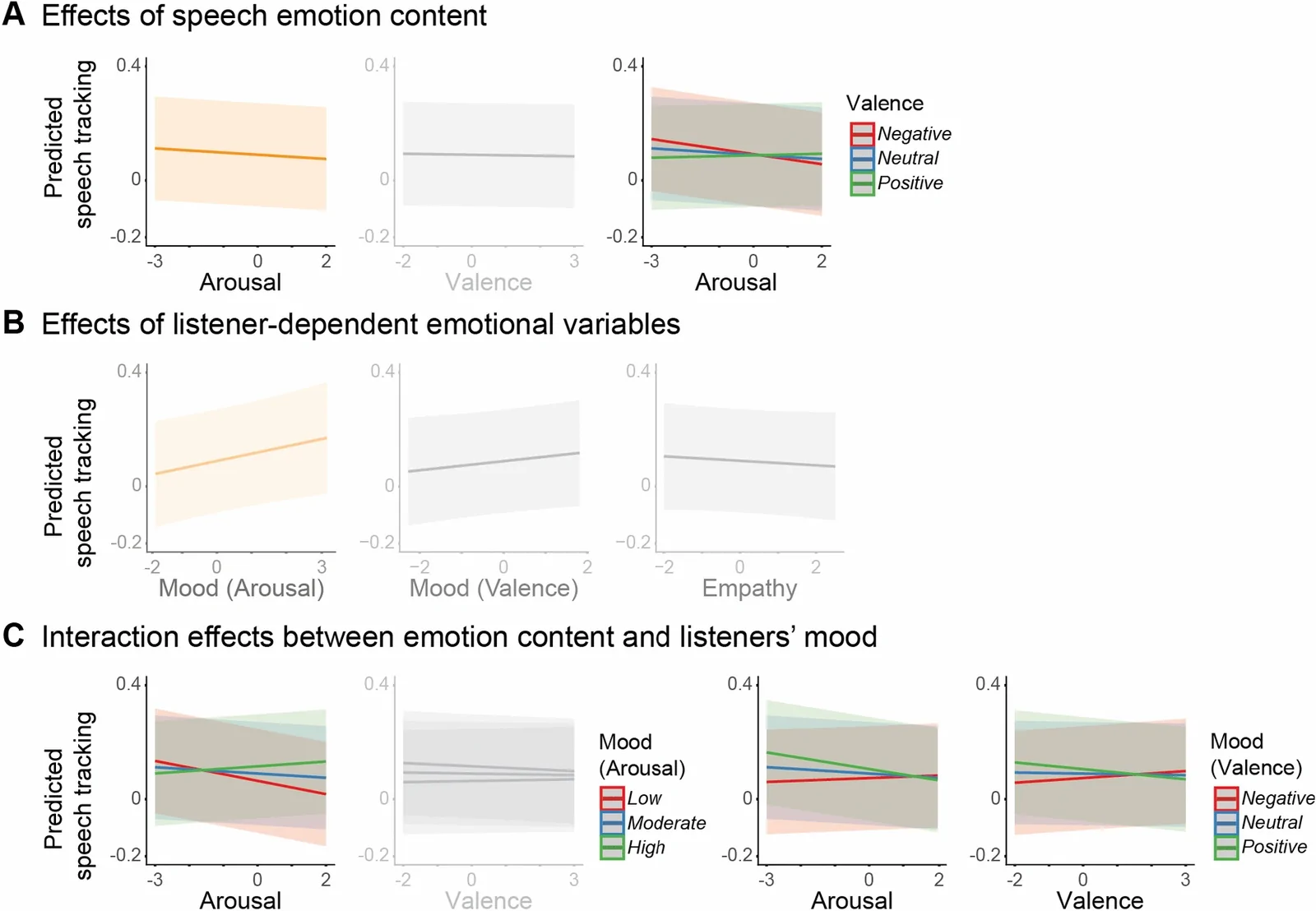
Relationship between hEOG tracking of speech and emotion-related predictors. Each plot displays the predicted MI values from the model (y-axis) as a function of the corresponding predictor (x-axis), with shaded areas representing 95% confidence intervals. Variables that did not predict hEOG envelope tracking are shown in gray; predictors that did not survive FDR correction are shown in paler colours. (**A**) Effects of subjective emotion ratings of speech on hEOG speech tracking. The model revealed significant main effects of arousal on hEOG tracking of speech signal. Speech valence was not a significant predictor; however, its interaction with speech arousal predicted hEOG tracking of speech. (**B**) Effects of listener-dependent emotional variables (arousal and valence dimensions of current mood and trait empathy) on hEOG speech tracking. The main effect of mood arousal did not survive FDR correction. Neither the main effect of mood on the valence dimension nor trait empathy predicted speech tracking. (**C**) Interactions between subjective emotion ratings of speech and listener-dependent emotional variables (arousal and valence dimensions of current mood) and their effect on hEOG speech tracking. The arousal dimension of mood significantly modulated the effects of speech arousal, whereas its interaction with speech valence was not a predictor of hEOG tracking. The model also revealed significant interaction effects between mood valence and both speech arousal and speech valence.

The interaction effect of *speech arousal* and *speech valence* was significant (*β* = 0.01, *p*_FDR_ <.001; peak effect observed at 340 ms in the single-lag models). Specifically, hEOG tracking was stronger for low-arousal speech when speech was perceived as more negative, whereas high arousal speech was tracked more strongly when speech was perceived as more positive (see Fig. 3A).

The model further revealed significant interaction effects between participants’ mood and emotional characteristics of speech (see Fig. 3C). When participants were in a low-arousal mood, low-arousal speech was tracked more strongly than high-arousal speech, in contrast, when participants were in a high-arousal mood, tracking was stronger for high-arousal than for low-arousal speech (*speech arousal* × *mood arousal*: *β* = 0.02, *p*_FDR_ < 0.001; peak effect observed at 820 ms in the single-lag models). Participants in a positive mood tracked low-arousal speech more strongly than high-arousal speech, whereas those in a negative mood exhibited the opposite pattern (*speech arousal* × *mood valence*: *β* = −0.01, *p*_FDR_ <.001; peak effect observed at 1300 ms in the single-lag models). In a positive mood, negative speech was tracked more strongly than positive speech, while in a negative mood, positive speech was tracked more strongly (*speech valence* × *mood valence*: *β* = −0.01, *p*_FDR_ <.01; peak effect observed at 640 ms in the single-lag models).

### Predictors of vEOG speech tracking

The full model predicting vEOG speech tracking revealed a significant main effect of *speech arousal* (*β* = −0.01, *p*_FDR_ <.001; peak effect observed at 1480 ms in the single-lag models), indicating stronger tracking for low- than high-arousal speech. The model also revealed a significant main effect of *speech valence* (*β* = −0.01, *p*_FDR_ =.01; peak effect observed at 160 ms in the single-lag models), with stronger tracking of negative speech than positive speech. The main effect of *HNR* on speech tracking in vEOG was significant (*β* = −0.02, *p*_FDR_ =.033, with higher *HNR* predicting lower tracking responses (see Table 3).

**Table 3:** Summary table for the mixed effects model predicting vEOG-speech MI values. A = Arousal dimension V = Valence dimension. Fixed-effect estimates (β) are reported with confidence intervals (95% CI) computed using the Wald method. p values were false discovery rate (FDR) corrected. Significant predictors are highlighted in bold. The model included random intercepts for participants, speech chunks and speech-eye lags. *p <.05 **p<.001.

Predictors	Estimates	95% CI		*p*_*uncorrected*_	*p*_FDR_
		LL	UL		
Intercept	0.20	−0.04	0.43	.100	.159
**Arousal**	−0.01	−0.02	−0.01	<.001**	<.001**
**Valence**	−0.01	−0.02	−0.004	.003*	.007*
Mood (A)	0.01	−0.02	0.05	.361	.494
Mood (V)	0.01	−0.02	0.04	.595	.637
Time-in-talk	0.02	−0.09	0.14	.702	.702
Trait empathy	−0.01	−0.04	0.02	.597	.637
Pitch	0.06	−0.13	0.24	.545	.637
F_1_	0.07	−0.09	0.24	.371	.494
**HNR**	−0.20	−0.35	−0.04	.014*	.033*
Talk	−0.43	−0.87	0.01	.056	.100
Arousal × Valence	0.01	0.001	0.01	.025*	.051
**Arousal × Mood (A)**	0.01	0.01	0.01	<.001**	<.001**
**Arousal × Mood (V)**	−0.01	−0.01	−0.004	<.001**	.003*
**Valence × Mood (A)**	−0.01	−0.02	−0.01	<.001**	<.001**
**Valence × Mood (V)**	0.02	0.01	0.02	<.001**	<.001**

**Fig. 4:**
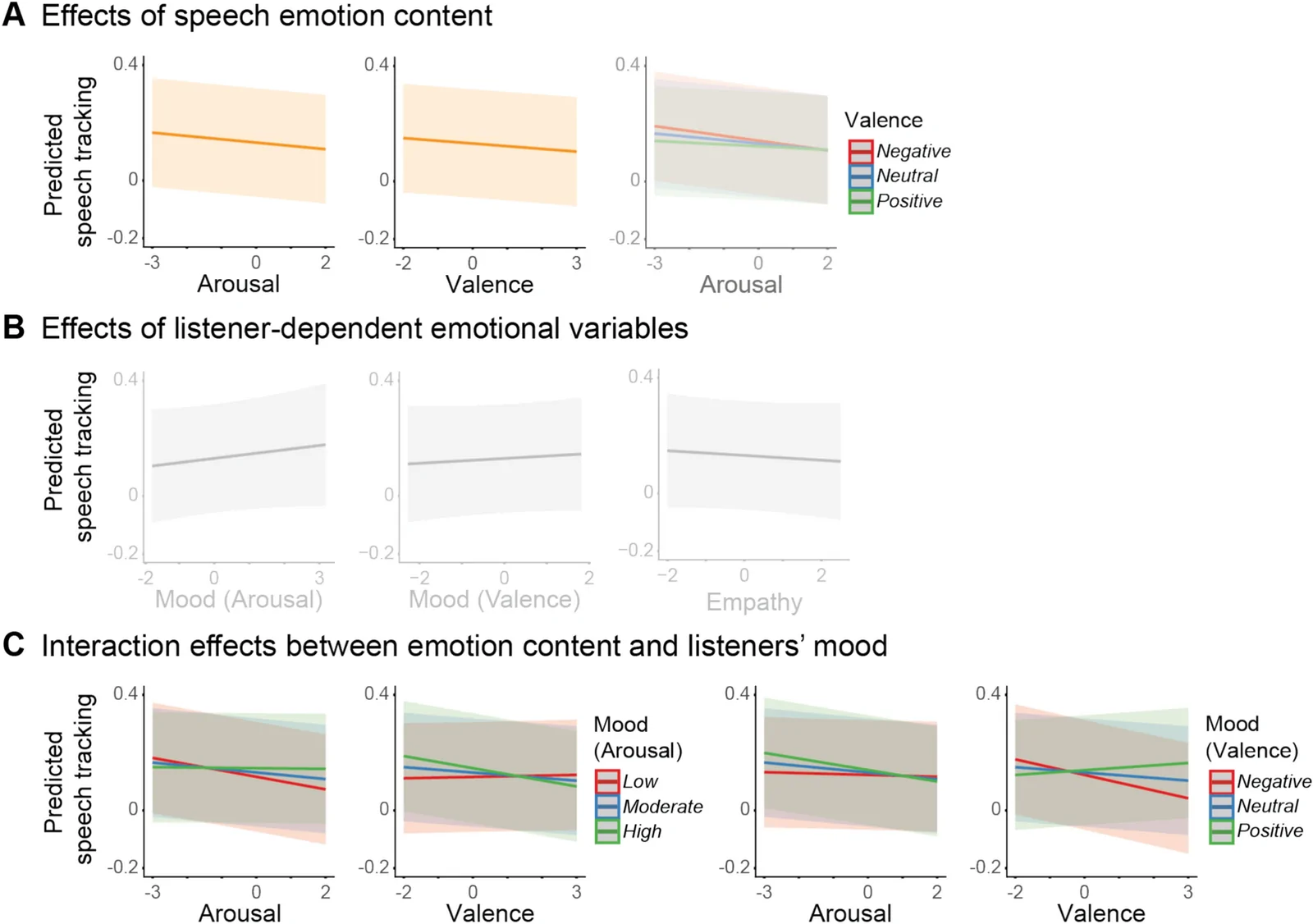
Relationship between vEOG tracking of speech and emotion-related predictors. Each plot displays the predicted MI values from the model (y-axis) as a function of the corresponding predictor (x-axis), with shaded areas representing 95% confidence intervals. Variables that did not predict vEOG envelope tracking are shown in gray; predictors that did not survive FDR correction are shown in paler colours. (**A**) Effects of subjective emotion ratings of speech on vEOG speech tracking. The model revealed significant main effects of arousal and valence on vEOG tracking of speech signal, as well as a significant interaction between the two. (**B**) Effects of listener-dependent emotional variables (arousal and valence dimensions of current mood and trait empathy) on vEOG speech tracking. The main effects of mood arousal, mood valence, and empathy on vEOG speech tracking were not statistically significant. (**C**) Interactions between subjective emotion ratings of speech and listener-dependent emotional variables (arousal and valence dimensions of current mood) and their effect on vEOG speech tracking. The model revealed significant interaction effects between mood dimensions and speech emotion content.

An interaction effect between *speech arousal* and *speech valence* predicted pupil speech tracking. However, this effect did not survive correction for multiple comparisons (p_uncorrected_ =.02; *p*_FDR_ =.051) (see Fig. 4A).

Significant interactions were observed between participants’ mood and speech emotions (see Fig. 4C). For mood arousal, participants in a low-arousal mood showed stronger tracking of low-arousal speech compared to high-arousal speech, whereas this pattern was not observed for those in a high-arousal mood (*speech arousal* x *mood arousal*: *β* = 0.01, *p*_FDR_ < 0.001; peak effect observed at 1540 ms in the single-lag models). Participants in a low-arousal mood also tracked positive speech stronger than negative speech, in contrast, participants in a high-arousal mood tracked negative speech stronger than positive speech (*speech valence* x *mood arousal: β* = −0.01, *p*_FDR_ < 0.001; peak effect observed at 880 ms in the single-lag models).

When participants were in a negative mood, tracking of negative speech was stronger than that of positive speech, whereas this pattern reversed for those in a positive mood, who showed stronger tracking of positive than negative speech (*speech valence* x *mood valence*: *β* = 0.02, *p*_FDR_ <.001; peak effect observed at 2020 ms in the single-lag models). Moreover, in a positive mood, the tracking of low-arousal speech was stronger than high-arousal speech, with this relationship is absent for negative mood (*speech arousal* x *mood valence*: *β* = −0.01, *p*_FDR_ =.003; peak effect observed at 700 ms in the single-lag models).

### Differences in emotion ratings between talks

To examine whether emotional responses differed across the two TED talks, we fitted separate linear mixed-effects models for arousal and valence ratings with Talk ID as a fixed effect, coded as a categorical variable with Talk 1 as the reference level. Random intercepts were included for participants and chunks to account for by-participant and by-chunk variability. Arousal ratings did not differ between the two talks (*p*_FDR_ =.997). In contrast, valence ratings were significantly more positive for Talk 2 compared to Talk 1 (*β* = 0.17, 95% CI [0.14–0.20], *p* <.001, *p*_FDR_ <.001). Chunk-level distributions of arousal and valence ratings are shown in Fig. 1C.

## Discussion

In the present study, we show that ocular dynamics (pupil dilation, horizontal and vertical EOG) track the quasi-rhythmic fluctuations of the speech envelope at low frequencies (0.5 - 12 Hz). Moreover, ocular speech tracking is sensitive to emotion-related factors, including emotional qualities embedded in the speech (as reflected by subjective ratings) and listener-dependent states, such as mood, and trait empathy.

### Pupil and eye movements significantly track speech at slow frequencies

Listeners showed reliable ocular tracking of the speech signal in our study, suggesting that bodily rhythms, much like neural rhythms, follow the temporal dynamics of continuous speech. Ocular speech tracking was observed at slow frequencies between 0.5 Hz and 12 Hz, with each eye-related signal showing somewhat different its frequency ranges. The specific frequency bands exhibiting significant tracking further varied across talks, which may be related to acoustic characteristics.

Previous studies have shown that vertical EOG activity, largely reflecting eye blinks, tracks higher-level linguistic structures, whereas horizontal EOG and pupil signals were not found to couple with speech features^20^. More recent gaze-based research, however, has demonstrated that both vertical and horizontal eye movements track the speech signal, with different tracking patterns (tracking of short sentences^21^:,tracking of continuous speech^24^:. Crucially, when we repeated the vEOG tracking analysis after removing blink segments, speech tracking persisted at low frequencies. This suggests that while blinks contribute to vEOG tracking, they are not the sole source of the effect, consistent with previous studies that excluded blink periods from the vEOG analysis^21,24^. Regarding pupil activity, in contrast to the findings by Jin et al.^20^, more recent work has shown that pupil responses align with word onsets and the speech envelope^23^. It is important to acknowledge that direct comparisons between these limited numbers of studies are challenging, given the variations in study designs, the specific speech features examined, and the methods to quantify speech tracking. Our findings support the presence of a coupling between eye-related physiological signals and the acoustic dynamics of continuous speech, providing further evidence for the precise temporal characteristics of this relationship. Importantly, we interpolated blinks and removed the canonical responses to saccades and blinks from the pupil signals^95^^,^^84^^,^^96^, thereby, ensuring that the observed pupil–speech coupling reflects processes distinct from eye movement related activity. The current dataset allows for complementary analyses of ocular speech tracking, such as temporal response function (TRF) modelling, which could be carried out using the publicly available data (https://osf.io/dsvzh/files/osfstorage).

We are still far from understanding the underlying mechanisms and functional relevance of ocular speech tracking. One proposed explanation is that it may serve as an active sensing mechanism, whereby motor actions are engaged to optimise perception, a notion suggested by predictive coding accounts^24,97^. The involvement of motor systems in speech perception has long been established^98,99^. The aforementioned growing body of literature adopting speech tracking measures extends this view by providing direct evidence for the involvement of eye movements in speech processing. Specifically, by aligning sensory systems with the incoming speech stream, eye movements could increase sensory gain and reduce uncertainty in dynamic listening environments^24^. While this perspective offers a promising framework, substantiating it will require systematic empirical work that integrates ocular, neural, and behavioural measures of speech processing.

### Subjective emotion ratings predict ocular tracking of speech

In line with our expectations, listeners’ subjective emotion ratings influenced the strength of ocular speech tracking, with distinct patterns across signals. The effect of valence followed a similar pattern for pupil and vertical tracking of speech: negative speech was associated with stronger speech tracking than positive speech. Note the effect of speech valence on pupil envelope tracking did not survive after FDR correction. However, the quadratic model (see Supplemental Materials) revealed a robust effect of the polynomial term of speech valence, suggesting stronger pupil tracking of neutral than positively or negatively valenced speech.

Valence is one of the most fundamental dimensions of emotional experience. A large body of findings show that both positive and negative linguistic stimuli hold processing advantages over neutral stimuli across diverse experimental designs^45,100,101^. However, it remains unclear whether stimuli at opposite ends of the continuum confer distinct advantages, and if so, in favour of which. Evidence suggests a processing advantage for positive words over neutral and negative words in visual lexical decision tasks^102–104^. A recent Bayesian meta-analysis also found a robust facilitative effect of positive valence on lexical decision times,effects of negative valence, however, were only observed occasionally, specifically when negative words were perceived as highly intense^105^. Some evidence suggests that valence effects appear to be non-linear, with a U-shaped pattern such that both positive and negative words are recognised faster than neutral words in an auditory lexical decision task^106^.

Emotional stimuli may bias attentional mechanisms to facilitate sensory processing^42,43^. In the auditory domain, electrophysiological studies suggest an enhanced spatial attention to negatively valence stimuli (i.e. threatening prosody), compared to positive valence (i.e. happy prosody) and neutral stimuli^107^. Behavioural evidence also suggests that temporal attention biases for angry prosody, in comparison to neutral prosody^40^. Our findings may be interpreted within this framework: negative speech may have captured listeners’ attention more effectively, leading to stronger alignment between ocular responses and the speech signal. While this interpretation aligns with previous evidence suggesting that increased attentional engagement enhances the strength of speech tracking^21^^,^^24^, which should be approached with caution given that speech stimuli used in the present study do not convey the same level of threat as the stimuli typically employed in the prior studies. Moreover, our study does not provide direct evidence for the role of attention in this relationship, and future studies are necessary to explore the interplay between emotion, attention, and ocular tracking in naturalistic paradigms.

The effects of arousal revealed distinct patterns for pupil and eye movement tracking of speech. For pupil responses, higher arousal was associated with stronger speech tracking, whereas both horizontal and vertical EOG signals tracked low-arousal speech stronger. Moreover, we observed a significant interaction between arousal and valence in predicting pupil and horizontal EOG tracking. The main effect pattern of arousal was repeated for the pupil tracking only in response to positive speech, which reversed for negative speech (i.e. low arousal led to stronger tracking in response to negative speech). For horizontal EOG, higher arousal enhanced tracking only when the speech was perceived as more positive. Overall, these results suggest that pupil and eye movements show distinct responses to high arousal speech. The sensitivity of pupil responses to arousal is well-established^31,66^, and there is some evidence for an enhanced pupil response to emotional speech^68^. Yet, pupil responses also index other cognitive processes, such as attention^108^, or listening effort^109^. While these processes cannot be disentangled in the present paradigm, future work should adopt experimental designs that isolate the contributions of attention, effort, and arousal more directly.

Our lag-based models further revealed that the influence of emotion-related predictors on ocular speech tracking unfolds over distinct temporal scales (see Supplementary Materials, Figure S3). Specifically, we observed an early effect of valence on vEOG speech tracking that peaked at around 160 ms and 100 ms, respectively, whereas the main effect of arousal consistently peaked at later lags (~1480-1780 ms) across all eye signals. The interaction between speech arousal and mood-related predictors also predominantly peaked at later time windows, typically between 1400 ms and 2200 ms. This temporal dissociation suggests that listeners’ judgements about stimulus valence (positivity or negativity) precede their arousal judgements in ocular speech tracking. Previous work has demonstrated that different cognitive and emotional processes are reflected at distinct temporal stages of the pupil response, spanning early and late components^23^. Extending these findings, here we show that both horizontal and vertical EOG signals exhibit similar multi-stage temporal dynamics during emotional speech tracking, suggesting that eye movements might also capture a mixture of fast and slower processes during continuous speech perception.

We also observed that lower HNR was associated with stronger pupil and vEOG tracking of the speech envelope. A similar trend was observed for hEOG tracking, although this did not survive FDR correction. Given that HNR is a measure of vocal clarity, less clear speech may be more effortful to process, requiring greater cognitive and attentional resources. This is consistent with the well-established link between pupil dilation and listening effort^76,108–110^, as well as more recent work showing that eye movements are sensitive to effortful speech listening^80^^,^^81^, extending these findings to the domain of speech tracking.

No previous study has directly examined how emotion modulates the tracking of continuous speech, whether at the physiological or cortical level. However, related evidence from research on infant-directed speech may offer an intriguing point of comparison. Infant-directed speech has been linked to enhanced cortical tracking in infant listeners with distinct synchronisation patterns relative to adult-directed speech^111–113^. Since infant-directed speech shares similar acoustic features with emotionally expressive speech, particularly its enhanced prosodic stress, it has been considered as an emotional form of speech^114^^,^^115^. Building on this resemblance, albeit speculatively, our findings may reflect a similar mechanism, whereby emotional speech facilitates temporal alignment to the acoustic envelope of speech in ocular responses.

A limitation of this study should be noted. While emotional variation in our study was captured through subjective ratings, it is also intrinsically linked to other stimulus properties, including semantic content, narrative structure, and speaker-specific delivery. Although we controlled for selected acoustic features, a substantial amount of stimulus-related variance remains unmodelled. Future work may focus on disentangling emotion-specific contributions from these potential sources of variance.

Another consideration is that the causal direction of this relationship remains unclear. While stronger tracking may occur because of perceiving the speech as more emotional, the reverse could also be plausible, where listeners perceive the speech as more emotional because they exhibit stronger tracking. Indeed, enhanced cortical tracking has been proposed as a mechanism underlying emotional induction in the music domain^116,117^, and empirical support for this account is growing^118^.

### Listener-dependent emotional factors modulate the relationships between emotion and speech tracking

The interaction patterns in our main models indicate that listeners’ mood shaped the relationship between speech emotions and envelope tracking, yet the nature of this modulation depended on the specific ocular signal. Across all signals, mood arousal interacted with speech arousal, and mood valence interacted with speech valence.

In high-arousal mood, high-arousal speech was associated with stronger tracking of the speech envelope in both the pupil and hEOG. Note that, for pupil dilation, the main effect of mood arousal also significantly predicted envelope tracking, that is listeners in high arousal mood showed overall strong tracking of speech. In low-arousal mood, low-arousal speech was associated with stronger tracking in both vEOG and hEOG, while this pattern was absent for pupil tracking. Mood arousal occasionally interacted with speech valence; in high-arousal mood, the tracking of negative speech was stronger than that of positive speech in both the pupil and vEOG, whereas this relationship was weaker in low arousal mood.

For the interaction between mood valence and speech valence, the patterns differed across ocular signals, with opposing effects for vEOG and hEOG, and a relatively weaker effect for pupil tracking. For listeners in a negative mood, negative speech was associated with stronger tracking in both the pupil and vEOG, whereas in hEOG positive speech led to stronger tracking than negative speech. In positive mood, pupil and hEOG tracking of negative speech was stronger. In contrast, vEOG showed the opposite pattern, with positive speech being associated with stronger tracking than negative speech. Moreover, significant interactions between mood valence and speech arousal were observed for eye-movement–based tracking. In both vEOG and hEOG, low-arousal speech was associated with stronger tracking in positive mood; in negative mood, this pattern reversed for hEOG, and no relationship between tracking and arousal observed for vEOG.

Many researchers stress the fundamental role of mood in information processing across domains, including language processing^119,120^. There is a general understanding of how positive or negative mood could influence the style of information processing. Specifically, positive mood is thought to promote a broader engagement with stimuli, whereas negative mood is linked with narrower, and more stimulus-oriented processing^121,122^. A congruency bias has also been suggested, such that stimuli with a valence that matches one’s emotional state receive processing advantages^123^. Our results are partially consistent with a congruency bias. In high-arousal mood, high-arousal speech was associated with stronger tracking, and the same held for low-arousal speech in low-arousal mood. However, it was less straightforward for mood valence: while congruency effects were evident in pupil and vEOG tracking, it did not apply to hEOG tracking of the speech envelope. Overall, our findings suggest a complex interaction between the emotional qualities of the speech and the listener’s mood, with effects that partly differed across ocular measures, highlighting the need for further research to elucidate these relationships in naturalistic listening contexts.

Beyond mood-related effects, participants with higher trait empathy showed weaker pupil tracking of the speech envelope. We interpret this finding as reflecting a shift in attentional focus: individuals with higher affective empathy may allocate more attention to the emotional content of speech rather than to its acoustic properties (i.e., the envelope), resulting in weaker pupil-speech coupling. Conversely, individuals with lower empathy may rely more heavily on acoustic cues when processing speech, leading to stronger envelope tracking. This interpretation is consistent with previous evidence linking higher empathy to increased sensitivity to emotional cues in social contexts^124–128^. Notably, this effect was not observed for speech tracking in eye movements, suggesting that the influence of empathy on speech tracking might operate through a specific pupil-arousal axis, rather than manifesting in eye activity in general. Here, we measure empathy as a unidimensional construct, future research could examine whether affective and cognitive components of empathy differentially modulate speech tracking at cortical and physiological levels.

## Conclusion

In conclusion, our first study demonstrated that the emotional content of naturalistic speech is dynamic, with individual speech chunks varying considerably in their perceived arousal and valence, confirming that our speech material elicits a rich and temporally varying emotional experience. In our second study, we showed that eye signals, including pupil dilation as well as vertical and horizontal EOG activity, tracked speech at low frequencies. This finding supports the view that ocular responses may act as an active sensing mechanism that contributes to speech processing even in the absence of visual input. We also found that subjective emotion ratings, and their interactions with listeners’ mood, modulated the tracking of speech signals, with distinct patterns for each eye signal. Together, our findings highlight the importance of considering both the emotional characteristics of the speech stimuli and listener-dependent factors, such as transient mood states and empathy, in speech tracking research, which has typically relied on neutral or positive speech materials.

## Supplementary Information

Supplementary Information.

## Data Availability

Data and stimuli are publicly available on the OSF (https://osf.io/dsvzh/files/osfstorage).
