# Supplementary material for: Pupil- and gaze dynamics track emotion content in naturalistic speech
Source: Sci Rep. 2026 Jun 18;16:27855. doi: 10.1038/s41598-026-57256-0 (PMC13547293; doi:10.1038/s41598-026-57256-0)
Supplement: Supplementary file 1 [file 41598_2026_57256_MOESM1_ESM.docx]

**Supplementary Materials**

1. **Chunk-level distributions of arousal and valence ratings**

In Study 1, we showed that participants’ arousal and valence ratings varied across individual speech chunks within each talk. Here, we provide chunk-level ridge plots to illustrate how emotion ratings fluctuate across the full sequence of chunks within each talk.

**Figure S1. Ridgeplots with distribution of emotion ratings for both talks.** Each horizontal line (i.e. y-axis) represents the density of ratings (arousal left, valence right) for a single speech chunk. x axis represent emotional arousal (between 0 and 100) and valence (between -100 and 100) ratings. Colouring reflects the rating level: red indicates mid-range values, blue reflects high arousal or positive valence, and green reflects low arousal or negative valence. Black dots indicate the mean value for each chunk.

Both perceived arousal and valence vary over time, although valence does more so than arousal. Notably, both talks include sections that are perceived as positive and sections that are perceived as negative. This confirms the suitability of the material for using it to study emotion-related ocular tracking responses.

2. **Acoustic feature extraction**

In study 1, we quantified the acoustic characteristics of each speech chunk by extracting features with the openSMILE toolkit (Eyben et al., 2010) in Python. We applied the extended Geneva Minimalistic Acoustic Parameter Set (eGeMAPS; (Eyben et al., 2015), which includes standardised parameters commonly used for affective speech processing. This extraction yielded 88 acoustic features per speech chunk, belonging to either frequency, energy, spectral, or temporal domains.

To reduce dimensionality and address multicollinearity among predictors, we performed a Principal Component Analysis (PCA) with varimax rotation on the full set of extracted features (Jolliffe & Cadima, 2016). We retained the first two principal components, which explained 39% and 11% of the variance, respectively, resulting in a cumulative explained variance of approximately 50%. We then selected the five acoustic features with the highest absolute loadings from each component for use as predictors in subsequent models (see Kamiloğlu et al., 2020 for a similar approach). These ten features reflected both core prosodic attributes (e.g., pitch variability, loudness, jitter) and spectral shape characteristics (e.g., Mel-Frequency Cepstral Coefficients (MFCC), spectral slope). Table 1 provides a list of the selected features and their factor loadings.

|  | Acoustic feature | Group | Factor loadings |
| --- | --- | --- | --- |
| Component 1 | F_3_ relative energy (M) | Spectral | .95 |
|  | F_2_ relative energy (M) | Spectral | .95 |
|  | F_1_ relative energy (M) | Spectral | .91 |
|  | MFCC_4_ (M) | Spectral | .85 |
|  | Loudness (M) | Temporal | .84 |
| Component 2 | F_0_ (SD) | Frequency | .85 |
|  | Harmonics-to-noise ratio (M) | Energy | .74 |
|  | Alpha ratio (SD) | Spectral | .71 |
|  | Jitter (M) | Frequency | .69 |
|  | Spectral slope (M) | Spectral | .69 |

**Table S1**. Acoustic features with highest loadings on the first two components. F_1_, F_2_. F_3_: the ratio of the energy of the spectral harmonic peak at the first, second, third formant’s centre frequency to the energy of the spectral peak at F_0_. MFCC_4_ (M): Arithmetic mean of the fourth MFCC across all frames, reflecting midlevel spectral contour. Loudness (M): Arithmetic mean of perceived intensity values computed from an auditory weighted Melband spectrum. F₀ (SD): Standard deviation of the fundamental frequency across voiced frames, indicating pitch variability. Harmonics-to-Noise Ratio (M): Arithmetic mean of the ratio between harmonic energy and noise energy, indicating voice periodicity. Alpha Ratio (SD): Standard deviation of the ratio between summed spectral energy, capturing variability in low vs high frequency balance. Jitter (M): Arithmetic mean of cycle-to-cycle period length deviations, reflecting glottal vibration irregularity. Spectral Slope (M): Arithmetic mean of the linear regression slope of the log power spectrum. Computational details and the relevance of each parameter are provided in Eyben et al. (2015).

3. **Relationship between subjective emotion ratings and acoustic features**

To examine whether acoustic features predicted subjective ratings of emotions, we fitted Linear Mixed-Effects Models (LME) in R (v4.4.2; *R Core Team*, 2024) using the *lme4* (Bates et al., 2015). The significance of fixed effects was evaluated using the *lmerTest* package (Kuznetsova et al., 2017), which applies Satterthwaite’s approximation to estimate degrees of freedom. To control for multiple comparisons, *p* values were adjusted using the false discovery rate (FDR) (Benjamini & Hochberg, 1995). The significance of main and interaction effects was further assessed using likelihood-ratio tests comparing nested models with and without the fixed effects of interest (Gelman & Hill, 2007). Model parameters were estimated using an unrestricted maximum likelihood optimisation procedure. All continuous predictors were standardised (z-scored) prior to model fitting. Assumptions for multilevel analyses (linearity, homogeneity of variance, multicollinearity, and normality of residuals) were visually inspected via diagnostic plots. A significance level of α = 0.05 was set for all the models.

Initially, both models included the ten PCA-selected acoustic predictors as fixed effects, and arousal and valence as outcome variables. Since the formant frequency measures showed severe collinearity (VIF > 30), the F_2_ and F_3_ variables were removed from the models. After this step, multicollinearity among the remaining predictors fell within acceptable limits (VIF < 6). Loudness was also excluded from the acoustic model because the stimuli presented to participants had been amplitude-normalised, which eliminated meaningful variation in loudness. The final model was:

$$Emotion Ratings \sim F0 + F1 + MFCC4 +$$

$$HNR +Alpha Ratio+Jitter+Spectral Slope+$$

$$\left( 1 \right| participant ID)+(1 | chunk ID)$$

For valence, the full model provided a significantly better fit than the null model (χ²(7) = 14.76, *p* = .039), indicating that the acoustic predictors jointly accounted for variance in valence ratings. At the uncorrected level, HNR showed a negative association with valence (*β* = −0.25, 95% CI [−0.49, −0.01], *p* = .046), however this effect did not survive FDR correction (*p*_FDR_ = .370), and no other acoustic predictor reached significance.

For arousal, the full model did not provide a significantly better fit than the null model (χ²(7) = 12.48, *p* = .086). F_0_ variability (*β* = −0.09, 95% CI [−0.16, −0.01], *p* = .021) and F_1_ relative energy (*β* = −0.10, 95% CI [−0.17, −0.02], *p* = .011) were both negatively associated with arousal ratings, suggesting that speech with greater pitch variability and higher F_1_ relative energy tended to be rated as less arousing. However, neither effect survived FDR correction (both *p*_FDR_ = .083).

4. **Device differences for pupil envelope tracking**

To assess whether the use of different eye-tracker devices influenced acoustic envelope tracking in the pupil signal or its relationship with emotion-related parameters, we fitted a linear mixed-effects model. This model was identical to the full envelope-tracking models, except that device type was added as a fixed effect. The analysis showed that device type did not predict MI values between the pupil signal and the speech envelope, and the pattern of significant predictors remained mostly unchanged relative to the full model (i.e., the model without device type)(see Table S2). This indicates that hardware differences did not influence our results for pupil-speech coupling.

| Predictors | Estimates | 95% CI | | *p_uncorrected_* | *p*_FDR_ |
| --- | --- | --- | --- | --- | --- |
|  |  | LL | UL |  |  |
| Intercept | 0.12 | -0.11 | 0.35 | .32 | .42 |
| **Arousal** | 0.02 | 0.01 | 0.02 | **<.oo1**** | **<.oo1**** |
| Valence | -0.01 | -0.01 | -0.001 | **.03*** | .06 |
| Mood (A) | 0.03 | 0.004 | 0.06 | **.03*** | .06 |
| Mood (V) | 0.01 | -0.02 | 0.04 | .60 | .60 |
| Time-in-talk | 0.02 | -0.08 | 0.14 | .57 | .60 |
| **Trait empathy** | -0.03 | -0.06 | -0.01 | **.02*** | **.048*** |
| Pitch | 0.05 | -0.11 | 0.22 | .53 | .60 |
| F_1_ | 0.06 | -0.09 | 0.21 | .45 | .55 |
| **HNR** | -0.20 | -0.35 | -0.06 | **.01*** | **.025*** |
| Talk | -0.37 | -0.78 | 0.03 | .07 | .12 |
| Device | 0.03 | -0.02 | 0.09 | .28 | .39 |
| **Arousal × Valence** | 0.02 | 0.02 | 0.03 | **<.001**** | **<.001**** |
| **Arousal × Mood (A)** | 0.01 | 0.01 | 0.02 | **<.001**** | **<.001**** |
| Arousal × Mood (V) | -0.005 | -0.01 | 0.001 | .09 | .13 |
| **Valence × Mood (A)** | -0.01 | -0.02 | -0.01 | **<.001**** | **<.001**** |
| Valence × Mood (V) | -0.005 | -0.01 | -0.000 | **.03*** | .06 |

**Table S2.** *Summary table for control model predicting pupil-speech MI values.* LL = Lower limit, UL = Upper limit, A = Arousal dimension, V = Valence dimension. Fixed-effect estimates (β) are reported with confidence intervals (95% CI) computed using the Wald method. *p* values were false discovery rate (FDR) corrected. Significant predictors are highlighted in bold. Blue p-values indicate effects that were significant in the full model without device as a predictor*.* The model included random intercepts for participants, speech chunks, and speech-eye lags. **p* <.05*, **p* <.001

5. **Time-resolved model results**

To estimate the temporal evolution of effects across the range of lags, we fitted separate models for each individual speech-eye lag (39 in total, between 100 and 2260 ms). The outcome variable was the mutual information between the speech signal and eye signals. The model structure was identical to those in main model: fixed effects were emotion ratings (i.e. speech arousal and speech valence) and participants’ mood scores (i.e. mood arousal and mood valence), as well as the interaction between emotion ratings and mood scores. We further entered chunk order, participant’s empathy scores, acoustic predictors (pitch, F_1_, and HNR) and talks as fixed effects into the models. Random factors included participants and speech chunks. These models allowed us to observe the temporal evolution of effects and to identify the time points at which the peak effects (i.e., the highest t-value) occurred. All continuous variables were z-scored.

**Figure S2. t-statistic curves across lags for each signal.** (A) Main model results for pupil tracking of the speech signal revealed a positive, significant effect of speech arousal with its influence on pupil tracking with a peak at approximately 1780 ms. While the effect of speech valence did not significant after FDR correction, this negative effect reached its peak at around 160 ms. The significant interaction between speech arousal and speech valence, which reached its peak at approximately 880 ms. The interaction effects between arousal and mood arousal (peak effect at 2020 ms); valence and mood arousal (peak effect at 2260 ms); valence and mood valence (not significant after FDR; peak at 1720) were also observed. (B) For hEOG tracking of the speech signal, the main effect of arousal was significant (peak effect at 1600 ms). The interaction between speech arousal and speech valence (peak effect at 340 ms); arousal and mood valence (peak effect at 1300 ms); valence and mood valence (peak effect at 640 ms); and between arousal and mood arousal (peak effect at 820 ms) were significantly predicted hEOG tracking. (C) The significant predictors of vEOG tracking were the main effects of speech arousal (peak effect at 1480 ms), speech valence (peak effect at 160 ms), as well as the interaction effect between valence and mood valence (peak effect at 2020 ms), arousal and mood valence (peak effect at 700 ms), arousal and mood arousal (peak effect at 1540 ms), valence and mood arousal (peak effect at 880 ms).

6. **Results for emotion models with quadratic term of valence**

We initially included both the first (linear) and second (quadratic) degree of the polynomial term for valence ratings in the model to capture potential nonlinear effects. These models revealed several main and interaction effects and were overall better fit than models with only linear valence. However, the inclusion of the quadratic term introduced complex interaction patterns, obscuring a clear interpretation. For clarity and parsimony, we opted to report the model with only linear component of valence in the main text. Here we present the result for quadratic models.

6.1. **Predictors of pupil speech tracking**

We observed a positive main effect of *speech arousal* in the main model (*β* = 0.04, *p*_FDR_ < .001), suggesting that high arousal speech was tracked stronger than low arousal speech. For *speech valence*, the linear term did not significantly predict pupil-speech tracking, however the quadratic term was significant (*Valence_Linear_*: *β* = -0.004, *p*_uncorrected_ = .21_,_ *p*_FDR_ = .30 ; *Valence_quadratic_*: *β* = -0.02, p_uncorrected_ < .001, *p*_FDR_ < .001). This suggests that speech tracking is stronger for neutral speech than valenced (positive or negative) speech. The main effect of *HNR* was significant (*β* = -0.20, *p*_FDR_= .02) and *empathy* was significant (*β* = -0.03, *p*_uncorrected_ = .03), however the effect of empathy did not remain significant after FDR correction (*p*_FDR_ = .06). No significant main effects were observed for the remaining predictors (see Table S3).

| Predictors | Estimates | 95% CI | | *p*_uncorrected_ | *p*_FDR_ |
| --- | --- | --- | --- | --- | --- |
|  |  | LL | UL |  |  |
| Intercept | 0.18 | -0.03 | 0.40 | .09 | .14 |
| **Arousal** | 0.04 | 0.03 | 0.04 | < .001** | **<.001**** |
| Valence (L) | -0.004 | -0.01 | 0.003 | .21 | .30 |
| **Valence (Q)** | -0.02 | -0.02 | -0.01 | <.001** | **<.001**** |
| Mood (A) | 0.01 | -0.02 | 0.04 | .51 | .57 |
| Mood (V) | -0.02 | -0.05 | 0.01 | .25 | .34 |
| Time-in-talk | 0.03 | -0.08 | 0.14 | .57 | .57 |
| Trait empathy | -0.03 | -0.06 | -0.004 | .03* | .06 |
| Pitch | 0.05 | -0.12 | 0.22 | .54 | .57 |
| F_1_ | 0.06 | -0.09 | 0.21 | .45 | .56 |
| **HNR** | -0.20 | -0.35 | -0.04 | .01* | **.02*** |
| Talk | -0.37 | -0.77 | 0.04 | .08 | .13 |
| **Arousal × Valence (L)** | 0.02 | 0.01 | 0.02 | <.001** | **<.001**** |
| **Arousal × Valence (Q)** | -0.01 | -0.01 | -0.004 | <.001** | **<.001**** |
| Arousal × Mood (A) | -0.002 | -0.01 | 0.004 | .55 | .57 |
| **Arousal × Mood (V)** | -0.03 | 0.03 | -0.02 | <.001** | **<.001**** |
| **Valence (L)× Mood (A)** | -0.01 | -0.02 | -0.01 | <.001** | **<.001**** |
| **Valence (Q)× Mood (A)** | 0.03 | 0.02 | 0.03 | <.001** | **<.001**** |
| Valence (L)× Mood (V) | -0.01 | -0.01 | -0.000 | .03* | .06 |
| **Valence (Q)× Mood (V)** | 0.03 | 0.03 | 0.03 | <.001** | **<.001**** |

**Table S3.** *Summary table for main model predicting pupil-speech MI values.* LL = Lower limit, UL = Upper limit, L = Linear term, Q = Quadratic term, A = Arousal dimension, V = Valence dimension. Fixed-effect estimates (β) are reported with confidence intervals (95% CI) computed using the Wald method. *p* values were false discovery rate (FDR) corrected. Significant predictors are highlighted in bold. Blue p-values indicate effects that were also significant in the linear-only model*.* The model included random intercepts for participants and speech chunks. **p* <.05*, **p* <.001

The main model also revealed several interaction effects. First, we found a two-way interaction effect of *speech arousal* × *speech valance*. This suggests that when speech arousal is rated high, positive speech is tracked stronger than negative speech (*speech arousal* × *speech valance*_linear_: *β* = 0.02, *p*_FDR_ < .001), but neutral speech is tracked stronger than both negative and positive speech (*speech arousal* × *speech valance*_quadratic_: *β* = -0.01, *p*_FDR_ < .001). When speech arousal is low, the relationship between valence_linear_ and speech tracking flips, and negative speech is tracked stronger than positive speech.

Second, we found interaction effects between speech emotion (i.e. arousal or valence ratings) and individuals’ own mood (both arousal and valence dimensions). When participants were in a negative mood, they tracked highly arousing speech stronger than low arousing speech, while this relationship was absent for people in a positive mood (*speech arousal × mood valence*: *β* = -0.03, *p*_FDR_ < .001). Additionally, people in a positive mood tracked negative speech more strongly than positive speech (*speech valence_linear_ × mood valence*: *β* = -0.01, *p*_uncorrected_ = .03, *p*_FDR_ = .06) and showed overall stronger tracking of emotional speech than neutral speech (*speech valence_quadratic_ × mood valence*: *β* = 0.03, *p*_FDR_ < .001). In contrast, participants in a negative mood tracked neutral speech stronger than emotional speech.

The arousal dimension of participants’ mood scores only interacted with speech valence (not with speech arousal) to predict pupil speech tracking. Participants who reported high-arousal mood scores showed stronger tracking of negative speech than positive speech (*speech valence_linear_ ×* *mood arousal: β* = -0.01, *p*_FDR_ < .001) and enhanced tracking of emotional speech compared to neutral speech (*speech valence_quadratic_ ×* *mood arousal: β* = 0.03, *p*_FDR_ < .001). Participants in a low-arousal mood tracked neutral speech more strongly than emotional speech.

6.2. **Predictors of hEOG speech tracking**

The main model with hEOG-speech tracking as outcome revealed that only the main effect of *mood arousal* was significant (*β* = 0.03, *p*_uncorrected_ = .01, *p*_FDR_ = .04), suggesting participants in high arousal mood showed overall stronger tracking of speech. None of the remaining predictors showed a significant main effect (see Table S4).

| Predictors | Estimates | 95% CI | | *p*_uncorrected_ | *p*_FDR_ |
| --- | --- | --- | --- | --- | --- |
|  |  | LL | UL |  |  |
| Intercept | 0.18 | -0.04 | 0.40 | .11 | .53 |
| Arousal | -0.01 | -0.01 | 0.00 | .09 | .17 |
| Valence (L) | -0.003 | -0.01 | 0.00 | .42 | .53 |
| Valence (Q) | -0.01 | -0.01 | 0.00 | .05 | .12 |
| **Mood (A)** | 0.03 | 0.01 | 0.06 | .01* | **.04*** |
| Mood (V) | 0.01 | -0.01 | 0.03 | .43 | .53 |
| Time-in-talk | 0.02 | -0.09 | 0.14 | .67 | .73 |
| Trait empathy | -0.01 | -0.03 | 0.02 | .56 | .62 |
| Pitch | 0.03 | -0.14 | 0.21 | .71 | .53 |
| F_1_ | 0.11 | -0.05 | 0.26 | .18 | .64 |
| HNR | -0.17 | -0.32 | -0.02 | .03* | .07 |
| Talk | -0.40 | -0.82 | 0.02 | .44 | .53 |
| **Arousal × Valence (L)** | 0.01 | 0.01 | 0.02 | <.001** | **<.001**** |
| Arousal × Valence (Q) | 0.003 | -0.00 | 0.01 | .11 | .19 |
| **Arousal × Mood (A)** | 0.02 | 0.02 | 0.03 | <.001** | **<.001**** |
| **Arousal × Mood (V)** | -0.02 | -0.03 | -0.01 | <.001** | **<.001**** |
| Valence (L)× Mood (A) | -0.004 | -0.01 | 0.00 | .08 | .16 |
| **Valence (Q)× Mood (A)** | -0.01 | -0.01 | -0.00 | <.001** | **<.001**** |
| **Valence (L)× Mood (V)** | -0.01 | -0.01 | -0.01 | <.001** | **<.001**** |
| **Valence (Q)× Mood (V)** | 0.01 | 0.00 | -0.01 | .002* | **.006*** |

**Table S4.** *Summary table for the mixed effects model predicting hEOG-speech MI value.* LL = Lower limit, UL = Upper limit, L = Linear term, Q = Quadratic term A = Arousal dimension V = Valence dimension. Fixed-effect estimates (β) are reported with confidence intervals (95% CI) computed using the Wald method. *p* values were false discovery rate (FDR) corrected. Significant predictors are highlighted in bold. Blue p-values indicate effects that were also significant in the linear-only model. The model included random intercepts for participants and speech chunks. **p <.05 **p<.001*

The interaction between *speech arousal* and *speech valence* was significant (*speech arousal × speech valence_linear_*: *β* = 0.01, *p*_FDR_ < .001). Specifically, hEOG tracking was stronger for negative speech when speech arousal is low, whereas for high-arousal speech, positive speech was tracked more strongly.

The model further revealed significant interaction effects between participants mood and emotional characteristics of speech. Participants in a negative mood tracked highly arousing and positive speech more strongly than low-arousing or negative speech, whereas those in a positive mood exhibited the opposite pattern (*speech* *arousal × mood valence*: *β* = -0.02, *p*_FDR_ < .001; speech *valence_linear_ × mood valence*: *β* = -0.01, *p*_FDR_ < .001). Additionally, neutral speech was tracked more strongly in a negative mood, while emotional speech was tracked more strongly in a positive mood (speech *valence_quadratic_ × mood valence*: *β* = 0.01, *p*_FDR_ = 0.007).

When participants were in a low-arousal mood, low-arousal speech was tracked more strongly than high-arousal speech, in contrast, tracking was stronger for high-arousal than for low-arousal speech in a high-arousal mood (*arousal × mood arousal*: *β* = 0.02, *p*_FDR_ < .001). Furthermore, while in a high-arousal mood neutral speech was tracked more strongly than emotionally valenced speech, the opposite was found in a low-arousal mood (*valence_quadratic_ × mood arousal*: *β* = -0.01, *p*_FDR_ < 0.001).

6.3. **Predictors of vEOG speech tracking**

In the main model predicting vEOG speech tracking, only the main effect of *valence* (*linear*: *β* = -0.01, *p*_uncorrected_ = .01, *p*_FDR_ = .04; *quadratic*: *β* = 0.01, *p*_uncorrected_ = .03, *p*_FDR_ = .07) was significant, suggesting that negative speech was tracked stronger than positive speech (see Table S5).

| Predictors | Estimates | 95% CI | | *p*_uncorrected_ | *p*_FDR_ |
| --- | --- | --- | --- | --- | --- |
|  |  | LL | UL |  |  |
| Intercept | 0.19 | -0.04 | 0.43 | .10 | .15 |
| Arousal | -0.01 | -0.01 | 0.00 | .05 | .11 |
| **Valence (L)** | -0.01 | -0.02 | -0.00 | **.01*** | **.04*** |
| Valence (Q) | 0.01 | 0.00 | 0.01 | **.03*** | .07 |
| Mood (A) | 0.02 | -0.01 | 0.05 | .16 | .21 |
| Mood (V) | 0.01 | -0.02 | 0.04 | .46 | .54 |
| Time-in-talk | 0.02 | -0.09 | 0.14 | .70 | .70 |
| Trait empathy | -0.01 | -0.04 | 0.02 | .61 | .64 |
| Pitch | 0.06 | -0.13 | 0.24 | .54 | .60 |
| F_1_ | 0.07 | -0.09 | 0.24 | .37 | .46 |
| **HNR** | -0.20 | -0.35 | -0.04 | .01* | **.04*** |
| Talk | -0.43 | -0.87 | 0.01 | .06 | .11 |
| Arousal × Valence (L) | 0.004 | -0.000 | 0.01 | .08 | .13 |
| **Arousal × Valence (Q)** | -0.01 | -0.01 | -0.00 | .003 | **.01*** |
| **Arousal × Mood (A)** | 0.02 | 0.01 | 0.02 | **<.001**** | **<.001**** |
| Arousal × Mood (V) | -0.01 | -0.01 | 0.00 | .09 | .14 |
| **Valence (L)× Mood (A)** | -0.01 | -0.02 | -0.01 | **<.001**** | **<.001**** |
| **Valence (Q)× Mood (A)** | -0.01 | -0.01 | -0.01 | **<.001**** | **<.001**** |
| **Valence (L)× Mood (V)** | 0.02 | 0.01 | 0.02 | **<.001**** | **<.001**** |
| Valence (Q)× Mood (V) | -0.004 | -0.01 | 0.000 | .06 | .11 |

**Table S5.** *Summary table for the mixed effects model predicting vEOG-speech MI values.* LL = Lower limit, UL = Upper limit, L = Linear term Q = Quadratic term A = Arousal dimension V = Valence dimension. Fixed-effect estimates (β) are reported with confidence intervals (95% CI) computed using the Wald method. p values were false discovery rate (FDR) corrected. Significant predictors are highlighted in bold. Blue p-values indicate effects that were also significant in the linear-only model. The model included random intercepts for participants and speech chunks. *p <.05 **p<.001

We found an interaction effect between speech arousal and speech valence (*arousal × valence_quadratic_*: *β* = -0.01, *p*_FDR_ = 0.01) This interaction effect suggests that emotionally valenced) speech elicited stronger vEOG tracking than neutral speech when the speech was perceived as low in arousal, whereas this pattern diminished for high-arousing speech.

Significant interactions were also observed between participants’ mood and speech emotions. When participants were in a negative mood, tracking of negative speech was stronger than that of positive speech, whereas this pattern reversed for those in a positive mood, who showed stronger tracking of positive than negative speech *(valence_linear_ x mood valence*: *β* = 0.02, *p*_FDR_ < .001).

For mood arousal, participants in a low-arousal mood showed stronger tracking of low-arousal speech compared to high-arousal speech, whereas the opposite pattern was observed for those in a high-arousal mood, who tracked high-arousal speech more strongly (*arousal x mood arousal*: *β* = 0.02, *p*_FDR_ < 0.001). Participants in a low-arousal mood also tracked positive speech more strongly than negative speech (*valence_linear_ x mood arousal β* = -0.01, *p*_FDR_ < 0.001) and showed stronger tracking of valanced speech compared to neutral speech (*valence_quadratic_ x mood arousal: β* = -0.01, *p*_FDR_ < 0.001).

7. **MI between eye signals and speech envelope**

Cluster-based permutation tests revealed significant speech tracking at low frequencies across all ocular signals. Figure S3 displays the observed and surrogate MI values for each signal.

**Figure S3.** Mutual information between the speech envelope and pupil dilation (A), hEOG (B), and vEOG (C) for Talk 1 (light green) and Talk 2 (dark green). Red lines represent mean surrogate MI values (N = 100) Shaded areas represent 95% confidence intervals.

8. **vEOG-Speech envelope tracking persists after blink removal**

We identified and removed the blinks from the vEOG signal using a custom-made MATLAB script built on the FieldTrip toolbox (Oostenveld et al., 2011). The pipeline combined threshold-based automatic detection with manual correction. Prior to detection, the vEOG signal was high-pass filtered at 1 Hz (3rd-order Butterworth) and demeaned to remove slow drifts. Blink detection was then applied to this filtered signal using an amplitude threshold of 40 µV, retaining only deflections with durations between 20 and 400 ms. Following automatic detection, each chunk was visually inspected and manually corrected by adding or removing blinks if necessary. Detected blink regions were removed and replaced via linear interpolation.

To test whether vEOG-speech coupling persisted after blink removal, we ran a MI analysis following the same procedure as in Study 2. Blink-cleaned vEOG continued to show significant speech envelope tracking in both talks. For Talk 1, we found a positive cluster between 0.53 and 3.69 Hz, (cluster *t*_sum_= 299.07, Cohen's d_Peak_ = 6.95, *p* = .001). For Talk 2, the cluster extended between 0.53 and 12.00 Hz (cluster *t*_sum_ = 340.77, Cohen's d_Peak_ = 6.71, *p* = .001) for the second talk. These results indicate that vEOG-speech envelope coupling is preserved even when blink-related activity is removed.

**Figure S4. Speech envelope tracking in blink-cleaned vEOG.** Panel A shows mutual information between the speech envelope and blink-cleaned vEOG across frequencies, separately for Talk 1 (left) and Talk 2 (right); real MI values are shown in light/dark green, surrogate MI values (*N* = 100) in red. Panel B shows cluster-corrected *t*-values for each talk. Even after blink removal, vEOG tracked the speech envelope at low frequencies in both talks.

**References**

Bates, D., Mächler, M., Bolker, B., & Walker, S. (2015). Fitting Linear Mixed-Effects Models Using lme4. *Journal of Statistical Software*, *67*, 1–48. https://doi.org/10.18637/jss.v067.i01

Benjamini, Y., & Hochberg, Y. (1995). Controlling the False Discovery Rate: A Practical and Powerful Approach to Multiple Testing. *Journal of the Royal Statistical Society: Series B (Methodological)*, *57*(1), 289–300. https://doi.org/10.1111/j.2517-6161.1995.tb02031.x

Eyben, F., Scherer, K. R., Schuller, B. W., Sundberg, J., André, E., Busso, C., Devillers, L. Y., Epps, J., Laukka, P., Narayanan, S. S., & Truong, K. P. (2015). The Geneva Minimalistic Acoustic Parameter Set (GeMAPS) for Voice Research and Affective Computing. *IEEE Transactions on Affective Computing*, *7*(2), 190–202. https://doi.org/10.1109/TAFFC.2015.2457417

Eyben, F., Wöllmer, M., & Schuller, B. (2010). Opensmile: The munich versatile and fast open-source audio feature extractor. *Proceedings of the 18th ACM International Conference on Multimedia, MM ’10*, 1459–1462. https://doi.org/10.1145/1873951.1874246

Gelman, A., & Hill, J. (2007). *Data Analysis Using Regression and Multilevel/Hierarchical Models*. Cambridge University Press.

Jolliffe, I. T., & Cadima, J. (2016). Principal component analysis: A review and recent developments. *Philosophical Transactions. Series A, Mathematical, Physical, and Engineering Sciences*, *374*(2065), 20150202. https://doi.org/10.1098/rsta.2015.0202

Kamiloğlu, R. G., Slocombe, K. E., Haun, D. B. M., & Sauter, D. A. (2020). *Human listeners’ perception of behavioural context and core affect dimensions in chimpanzee vocalizations*.

Kuznetsova, A., Brockhoff, P. B., & Christensen, R. H. B. (2017). lmerTest Package: Tests in Linear Mixed Effects Models. *Journal of Statistical Software*, *82*, 1–26. https://doi.org/10.18637/jss.v082.i13

Oostenveld, R., Fries, P., Maris, E., & Schoffelen, J.-M. (2011). FieldTrip: Open Source Software for Advanced Analysis of MEG, EEG, and Invasive Electrophysiological Data. *Computational Intelligence and Neuroscience*, *2011*(1), 156869. https://doi.org/10.1155/2011/156869

*R Core Team*. (2024). [Computer software].
